# Donut-like organization of inhibition underlies categorical neural responses in the midbrain

**DOI:** 10.1038/s41467-022-29318-0

**Published:** 2022-03-30

**Authors:** Nagaraj R. Mahajan, Shreesh P. Mysore

**Affiliations:** 1 Department of Electrical and Computer Engineering, Johns Hopkins University, Baltimore, MD USA; 2 Departments of Psychological and Brain Sciences, Johns Hopkins University, Baltimore, MD USA; 3 The Solomon H. Snyder Department of Neuroscience, Johns Hopkins University, Baltimore, MD USA

**Keywords:** Neuroscience, Neural circuits, Decision, Visual system

## Abstract

Categorical neural responses underlie various forms of selection and decision-making. Such binary-like responses promote robust signaling of the winner in the presence of input ambiguity and neural noise. Here, we show that a ‘donut-like’ inhibitory mechanism in which each competing option suppresses all options except itself, is highly effective at generating categorical neural responses. It surpasses motifs of feedback inhibition, recurrent excitation, and divisive normalization invoked frequently in decision-making models. We demonstrate experimentally not only that this mechanism operates in the midbrain spatial selection network in barn owls, but also that it is necessary for categorical signaling by it. The functional pattern of neural inhibition in the midbrain forms an exquisitely structured ‘multi-holed’ donut consistent with this network’s combinatorial inhibitory function for stimulus selection. Additionally, modeling reveals a generalizable neural implementation of the donut-like motif for categorical selection. Self-sparing inhibition may, therefore, be a powerful circuit module central to categorization.

## Abstract (web summary)

Decision making is facilitated by categorical neuronal responses, which robustly signal a winner despite input noise. In this study, the authors demonstrate that a donut-like inhibition motif effectively generates such categorical responses.

## Introduction

**Fig. 1:**
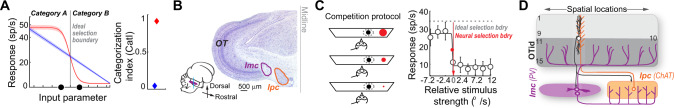
Categorical neural responses in the owl midbrain network. **A** Left: Schematic comparing two mathematically generated ‘neural’ response profiles, as a function of continuously varying input. Red—categorical response profile, blue—linear response profile. Gray vertical line: ideal selection (or categorization) boundary. Translucent band: variability in responses (s.e.m); fano factor of 6 used to generate these responses (Methods). Filled black dots: Two competing inputs just straddling the selection boundary: they are nearly equal but belonging to different categories, each at a distance of 3 units from the selection boundary. Right: Categorization index (CatI); characterizes strength of categorization of response profiles in left panel. CatI is insensitive to scaling of response means and accounts for response variability (Fig. S1A–C; Methods)^6,8^. **B** Midbrain selection network in barn owl. Inset: Side view of owl brain. Vertical blue line: line of section. Main: Coronal section through owl midbrain showing optic tectum (OT), isthmi pars magnocellularis (Imc, GABAergic, parvalbumin-positive, purple outline), and isthmi pars parvocellularis (Ipc, cholinergic, orange outline). **C** Left: Schematic of “competition protocol” used in (non-behaving) owls^9,22^. A stimulus of fixed strength (black dot) is presented inside the spatial receptive field (RF; dashed oval) of a neuron, while a competitor (red dot) of varying strength is presented far outside the RF. Strength of the stimuli is controlled by their loom speeds (°/s); denoted here by size of dots. Right: Categorical (“switch-like”) response profile of a neuron in the intermediate and deep layers of the OT (OTid) measured using the competition protocol in left panel. Red vertical line: neural selection boundary; indicates the relative strength at which neural responses switch from being at a high level to a low level. Gray vertical line: ideal selection boundary; indicates the relative strength of 0, at which the two stimuli are equally strong. The neural selection boundary nearly overlaps with ideal one. Data reproduced with permission^9^. **D** Schematic of connectivity within the avian midbrain selection network. Layers (1–15) of OT are shown. OTid: intermediate and deep layers (layers 11–15). Cholinergic Ipc neuron (orange circle) receives focal input from OT (black circle, layer 10), and send projections focally back to OT (orange projections). GABAergic Imc neuron (purple circles) receives focal input from OT (black circle, layer 10), and but sends inhibitory projections broadly across OTid as well as Ipc (purple projections). Consequently, Imc neurons suppress OTid responses via two pathways: (i) the direct pathway to the OTid^31^ (Imc→OT), and (ii) the indirect pathway that inhibits the potent point-to-point cholinergic amplifiers of OTid, namely the Ipc^31,33^ (Imc→ Ipc→ OT). See also Fig. S1. Source data are provided as a Source Data file.

Categorization, the transformation of continuously varying inputs into discrete output groups, is a fundamental component of perception and decision-making^1–3^. Neural responses that are explicitly categorical^4^, have been reported across brain areas and animal species in a variety of perceptual and decision-making contexts^2,5–13^. Such response profiles, which involve a large and abrupt change in firing rate across the category boundary (Fig. 1A-left, red), are computationally advantageous: they enhance downstream decoding of the selected category (or “winner”) particularly when competing options are similar (i.e., in the face of input ambiguity), and when neural responses are variable (i.e., in the face of representational uncertainty; Fig. 1A; red vs. blue)^9,14^. Despite the pervasiveness^2,5–13^ and computational benefit of such categorical neural response profiles, how the brain implements them is unknown. Specifically, it is unclear what identifiable circuit mechanisms are essential for producing categorical neural response profiles.

An excellent site in the brain at which to investigate neural circuit mechanisms of categorization is a vertebrate midbrain network that plays a causal role in controlling gaze and spatial attention^15–21^. This network, which encodes sensory space topographically, includes the optic tectum (called the superior colliculus, SC, in mammals) and several nuclei in the midbrain tegmentum, referred to as the isthmic nuclei^21^ (Fig. 1B). In the barn owl, this network has been shown to categorize stimuli into two categories: “highest priority” and “others”^8,9,22^, with the priority of a stimulus being defined as the combination of its physical salience and behavioral relevance^23^. This categorization manifests as “switch-like” responses in a subset of neurons in the intermediate and deep layers of the owl optic tectum (OTid, SCid in mammals)^8,9,22^. These neurons fire at a high rate when the stimulus inside their spatial receptive field (RF) is the highest priority, but switch abruptly to a lower firing rate when a distant, competing stimulus becomes the highest priority one (Fig. 1C; red neural selection boundary nearly overlaps with gray ideal selection boundary). Such switch-like responses markedly improve discriminability of the location of the highest priority stimulus among competing stimuli of similar priority^9,14^. Additionally, such categorization by OTid in owls accounts well for the specific pattern of spatial selection deficits observed in monkeys following inactivation of the SCid^14^: worsening of the impairment in selecting a target among distracters as they become more similar to the target^17,18,24^. Together, these studies support that categorical responses in OTid enhance reliable readout of the highest priority stimulus for gaze or spatial attention behavior, particularly when competing stimuli are of similar strength (or more generally, similar priority)^14,21,22^.

The responses of OTid neurons are regulated by two key isthmic nuclei in the midbrain selection network. OTid responses to single stimuli are enhanced multiplicatively by cholinergic neurons of the isthmi pars parvocellularis (Ipc), which exhibit point-to-point recurrent connectivity with the OT (Fig. 1D-orange)^25–27^. In parallel, responses of OTid neurons to multiple competing stimuli are controlled by inhibitory neurons of the isthmi pars magnocellularis (Imc) (Fig. 1D-purple)^28–31^. Imc neurons receive focal input from the OT but send long-range projections broadly across both the OTid and Ipc space maps, suppressing OT through the direct (Imc→OTid) pathway as well as the indirect (Imc→Ipc→OTid) pathway (Fig. 1D-purple projections). The Imc controls competitive interactions across the OTid space map: focal inactivation of Imc neurons abolishes all competitive interactions in the OTid (and Ipc)^32,33^. Despite these insights, how categorical neural response profiles in the OTid are generated is an open question.

Here, using a combination of neurally grounded computational modeling, dual electrophysiological recordings, and focal iontophoretic neural inactivation^32^, we investigate how categorical neural responses in the barn owl OTid are generated by computations in the OT-Imc-Ipc network. We demonstrate that a donut-like pattern of spatial inhibition in this network causally controls categorization by OTid, and also that this inhibition is implemented with an intricate multi-holed donut-like pattern across interconnected brain areas in support of categorization across space. These results suggest that donut-like inhibition may be a fundamental circuit motif for generating categorical neural responses, a function central to spatial selection, perception, and decision making^1,34^.

## Results

### Donut-like inhibitory motif emerges as a powerful mechanism for generating categorical responses in model circuits of the avian midbrain selection network

**Fig. 2:**
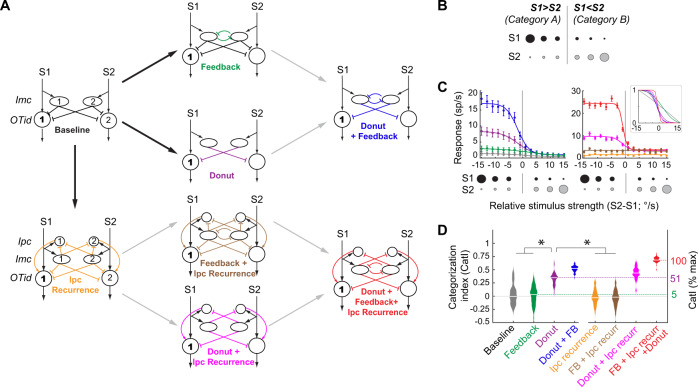
Donut-like inhibition surpasses other hypothesized circuit motifs for generating categorical responses in models of the avian midbrain selection network. **A** Computational models incorporating combinations of three different circuit motifs proposed in the literature as underlying categorical responses (Methods; Fig. S1). All models built upon generic “baseline” circuit (left column-top) capable of comparing competing options. Each model shows two “channels”; each channel is the group of neurons (numbered) involved in representing a stimulus (S1 or S2). Large circles: OTid neurons; ovals: Imc neurons; small circles: Ipc neurons. Arrows with pointed heads - excitatory connections, arrows with flat heads - inhibitory connections. The three circuit motifs are – feedback inhibition between competing channels (green; middle column - top), donut-like inhibition (self-sparing inhibition; purple; middle column-second from top; see also text), and recurrent amplification within each channel (through “Ipc” neuron; orange; left column-bottom). The goal of output neuron 1 (bold face) in each model is to signal if S1 > S2 (category “a”) or S2 > S1 (category “b”), when presented with S1 and S2 of varying relative strength following strength-morphing protocol. **B** Strength-morphing protocol (Methods). S1 and S2 are presented simultaneously “to” the model, S1(2) is inside the receptive field of neurons in channel 1 (2). As strength of S1 is decreased, that of S2 is systematically increased; strength of the stimuli is controlled by their loom speeds (°/s); denoted here by size of dots. Gray line: ideal selection boundary (when relative strength = 0). **C** Simulated response profiles of output neuron 1 from each of the models (colors) in A obtained using the strength- morphing protocol (bottom inset). Responses are mean ± s.e.m of 30 repetitions. The continuously varied input parameter was the relative strength of the two stimuli (S2-S1). Lines – best sigmoidal fits. Input-output functions of model neurons were sigmoids with Gaussian noise (Methods; fano factor=6). Right-Inset: Response profiles normalized between 0 and 1; only means are shown for clarity. **D** “Population” summary of CatI of response profiles from various circuit models (colors); *n* = 50 model neurons; center lines in the violin plots indicate median values. *p < 0.05, One-way ANOVA followed by Holm-Bonferroni correction for multiple paired comparisons; only a key subset of significant differences indicated for clarity. green vs. gray: *p* = 0.99, purple vs. gray: *p* = 5.98e-8, blue vs. purple: *p* = 5.98e-8, red vs. purple. See also Fig. S1. Source data are provided as a Source Data file.

As a first step in investigating the circuit mechanisms underlying categorical neural response profiles in the OTid, we turned to neurally grounded computational modeling. Starting with a simple model of the midbrain network capable of comparing the representations of competing stimuli^35–37^ (“Baseline” model; Fig. 2A, left column-top, oval “Imc” neurons deliver feedforward inhibition to circular “OTid” neurons; Methods), we introduced, systematically, each of three circuit motifs that have been proposed in the literature as potential mechanisms for generating categorical response profiles^38–41^. We compared directly the ability of these different circuit models to generate categorical neural response profiles.

The first mechanistic proposal from published literature is that feedback inhibition between the representations of competing options plays a key role in categorization. This is motivated by modeling studies of categorical decision-making^40,42^, work on direction selectivity in the retina^43^, as well as work on spatial selection in barn owls^44^. The reasoning is that the iterative nature of the feedback inhibition may allow for small differences in competing options to be amplified, resulting in large differences in steady-state competitive inhibition, and therefore, in neural responses. In the avian midbrain network, such feedback inhibition is known to be implemented as long-range reciprocal inhibitory projections among Imc neurons representing different (competing) options^31,44,45^. Therefore, in our model, we introduced feedback inhibition as reciprocal inhibition between the two model Imc neurons (Fig. 2A, middle-column top; green inhibitory connections)^44^.

The second mechanistic proposal is that recurrent excitation of the responses to each option plays a key role in categorization^40,46^. This is a common element in models of decision-making and is thought to aid categorical selection through response amplification^40,46^. In the avian midbrain network, recurrent excitation of OTid responses encoding for a particular spatial location is known to be implemented by the cholinergic Ipc neurons encoding for the same location^26^, resulting in focal multiplicative enhancement of OTid activity^40,46^ (Fig. 1D). Notably, as mentioned previously, Ipc neurons receive feedforward inhibition from Imc (Fig. 1D)^26,31^, as a result of which recurrent amplification of OTid by Ipc is regulated by Imc. Therefore, in our model, we introduced “Ipc recurrence” by incorporating amplifying Ipc neurons that lie downstream of Imc (Fig. 2A, left column-bottom; orange projections).

**Fig. 3:**
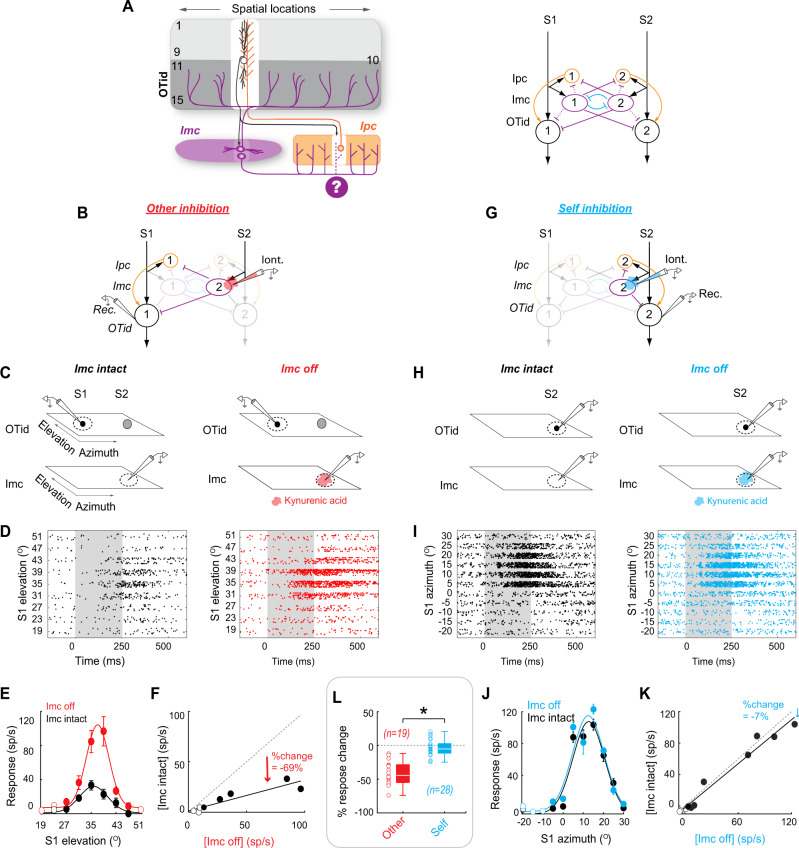
Barn owl midbrain selection network contains functional donut-like inhibitory motif. **A** Left: Schematic of avian (barn owl) midbrain selection network; modified from Fig. 1D; same conventions. Columns across layers of OT tissue, from left to right, encode individual locations in space (here, azimuth) topographically^92^. White column in OT space map: Inhibitory projections from Imc neuron that impinge broadly across OTid thought to spare this portion of OT which provides input to that Imc neuron (absence of purple projections here; white column surrounds the black OT10 neuron providing input^31^). Purple “?”: unknown if congruent portion of Ipc space map (white column) is also spared of Imc projections (dashed purple projections). Right: Network model showing the OT-Imc-Ipc circuit; conventions as in Fig. 2A. Dashed purple lines: Unknown if these “self”-inhibition connections exist functionally; in case of Imc→ Ipc, anatomical evidence is also lacking. **B**–**F** Measurement of the strength of net “other” inhibition from Imc → OTid with paired recordings in barn owl Imc and OTid. “Net” indicates the combined inhibition due to both the direct (Imc→ OTid) and indirect (Imc→ Ipc→ OTid) pathways (text). “Other” indicates that the OTid neuron encodes for (distant) spatial locations outside Imc neuron’s RF. **B** Experimental setup. Iontophoresis and recording electrode (Iont.) in the portion of Imc (encoding for stimulus S2); recording electrode (Rec.) in the portion of OTid encoding for distant location (and stimulus S1). Neurons and connections not immediately relevant to current experiment are shown ghosted-in. **C** Schematic of OTid and Imc space maps (quadrilaterals) showing RFs of neurons being recorded (dotted ovals) and stimulus protocol (black filled dot – S1; gray filled dot – S2). S1 and S2 are looming visual stimuli of fixed contrast but different loom speeds (strength^49^); S1 = 9.6 °/s, S2 = 19.2°/s (Methods). **D** Raster responses of example OTid neuron to paired stimulus protocol in (**C**), in the Imc-intact condition (left column; black data) and Imc-off condition (right column – red data). Imc inactivation by (reversible) iontophoresis of kynurenic acid (a pan-glutamate receptor blocker^32^; Methods). Gray shading: stimulus duration (**E**) Response firing rates of this OTid (computed from rasters in (**D**); spike count window = 150–350 ms); mean ± s.e.m (*n* = 12) Lines: best Gaussian fits. Filled dots: responses to S1 at locations inside the OTid RF; open circles, outside RF (Methods). **F** Scatter plot showing OTid responses in Imc-intact vs. Imc-off conditions. Line: Best fit straight line; slope = 0.31, r^2^ = 0.76. % change in responses = 100*(responses in Imc intact condition − responses in Imc off condition) / responses in Imc off condition = 100*(slope-1), directly estimates the strength of inhibition at this OTid neuron due to Imc (here, −69%; 100*(0.31–1); Methods). **G**–**K** Measurement of the strength of net “self” inhibition from Imc → OTid with paired recordings in Imc and OTid. (Conventions as in **B**–**F**). **G**, **H** OTid neuron is spatially aligned with Imc neuron (both encode overlapping locations); distance between OTid and Imc RF centers = 1.5°. **J** Response firing rates of this OTid (computed from rasters in I; spike count window = 75–350 ms); mean ± s.e.m (*n* = 15) Lines: best Gaussian fits. Filled dots: responses to S1 at locations inside the OTid RF; open circles, outside RF (Methods). **K** Line: Best fit straight line; slope = 0.93, R^2^ = 0.95. % change in responses directly estimates the strength of suppression at this OTid neuron due to Imc (here, −7%; Methods). **L** Population summary of strength of net “other” inhibition (red; *n* = 19 Imc-OTid pairs), and strength of net “self” inhibition (blue; *n* = 28 pairs) from Imc→ OTid. Average distance between OTid and Imc RF centers in “other” experiments = 26.8° +/− 2.3°; in “self” experiments = 2.9° +/− 0.7 °. *p = 7.8e-11 (red vs. blue), *p* = 9.43e-9 (red vs. 0), *p* = 0.12 (blue vs. 0), paired two-sided *t* tests with HBMC correction (Methods). Center lines in the box plots indicate median values, edges of the boxes indicate 25th and 75th percentile values, whiskers are at a distance of 1.5 times interquartile range from the edge of the box on respective sides. See also Fig. S2. Source data are provided as a Source Data file.

The third mechanistic proposal is that a donut-like pattern of competitive inhibition, i.e., one in which the representation of each option suppresses others more strongly than it suppresses itself, may play a key role in categorization. This is motivated by work in turtles^47^ as well as by the observation that categorical response profiles exhibit large response differences across the selection boundary. The reasoning is that having strong inhibition to “other” options, but weak “self”-inhibition can enhance response differences. In the avian midbrain network, inhibitory projections from each Imc neuron, which span the OTid space maps broadly (Fig. 1D), are thought to spare the portion of OT from which the Imc neuron receives input, suggesting an anatomical donut-like pattern of inhibition in the direct pathway from Imc to OTid^31^. Similar details about the indirect Imc→Ipc→ OT pathway are unknown. Therefore, in our model, we introduced donut-like inhibition by removing self-inhibitory connections in the direct Imc→OT pathway (Fig. 2A, middle column-second from top model; absence of purple projections from Imc neurons to aligned OTid neurons). (For completeness, in models that included Ipc recurrence, we also removed self-inhibitory connections in the indirect Imc→Ipc→OT pathway: Fig. 2A, middle column-bottom, absence of absence of pink projections from Imc neurons to aligned Ipc neurons; right column-bottom, absence of absence of red projections from Imc neurons to aligned Ipc neurons; see also Fig. 3 for direct experimental validation of this assumption).

The eight circuit models that arise from different combinations of these three proposed circuit motifs are illustrated in Fig. 2A. We next examined the ability of each model to produce categorical neural response profiles. We did so by measuring the responses of a designated output neuron in each model (Fig. 2A, “OTid” neuron 1) to a classic two-stimulus strength-morphing protocol^2,6,8^ (Fig. 2B). This protocol was identical to that used in past experimental work^8^, and involved the simultaneous presentation of two stimuli (S1 and S2) at distant spatial locations (or to distinct “channels” in the model). The relative strength of the S1 and S2 (controlled by their loom speed) was systematically varied, resulting in two well-defined stimulus strength-dependent categories^2,6,8^: S1 > S2 and S1 < S2; Fig. 2B; gray line). Model neurons in the circuit were simulated with noisy, sigmoidal input-output functions (30 repetitions per “neuron”, *n* = 50 “neurons”; Methods). The values of the parameters of these sigmoids, as well as the value of response fano factor used in the models (=6; Methods), were obtained from a series of previous experimental (electrophysiological) measurements of input-output functions in the barn owl midbrain network^9,28,30,44,48,49^.

To quantify the strength of categorization of the resulting response profiles, we used a generalized version of the classic categorization index used in the literature^6–8^. This index, defined as the average difference in responses between categories divided by the average difference in responses within categories, is insensitive to simple multiplicative scaling of the responses (Fig. S1A, B), and quantifies how “step-like” the response profiles are. Here, we modified it to define a generalized categorization index, “*CatI*”, which, in addition, takes into account neural response variability as well. *CatI* is defined as the average difference in discriminability (d’) between categories divided by the average difference within categories (Methods; Fig. 1A, right panel; Fig. S1A, B). We computed *CatI* for the strength-dependent response profiles from each of the eight models (Fig. 2A), and compared them statistically (Fig. 2D; ANOVA with HBMC correction; Methods).

Our simulation results revealed that feedback inhibition between the competing channels, reflecting inhibition between Imc neurons encoding for S1 and S2 in the avian midbrain network, had no significant effect on *CatI* (Fig. 2C-inset and 2D – green vs. gray; *p* = 0.99). This result was largely independent of the strength of feedback inhibition (Fig. S1D). Similarly, recurrent excitation within each channel, reflecting Ipc amplification of OTid activity in the avian midbrain network^25,27^, had no significant effect on *CatI* (Fig. 2C-inset and Fig. 2-orange vs. gray). This result as well was largely independent of the strength of recurrent amplification (Fig. S1E). Furthermore, the combination of feedback inhibition and recurrent excitation also had no significant effect on *CatI* (Fig. 2D-brown). These results held true also when we simulated an alternative implementation of recurrent excitation, one that has been used more generally in modeling work of decision-making^40,42^. In this variant, called just “recurrence” (as opposed to “Ipc recurrence”), the strength of amplification (Fig. S1F, top-left, orange arrow), is not regulated by competitive inhibition (from “Imc”; compare Fig. S1F, top-left to Fig. 2A, left column – bottom). Introduction of such recurrence, either by itself, or in conjunction with feedback inhibition, did not produce categorical responses (Fig. S1G, H).

By contrast, however, a donut-like pattern of inhibition from Imc to OTid in the model substantially boosted *CatI* of the response profiles (Fig. 2C-inset and 2D–purple vs. gray; *p* = 5.98e-8). The magnitude of improvement was inversely related to the strength of “self”- inhibition, reaching the maximum when self-inhibition was zero, i.e., when the pattern of inhibition was fully donut-like (Fig. S1I; *CatI*: *ρ* = −0.81, *p* = 2.6 e-3, Pearson correlation test). Whereas the presence of either or both recurrent excitation and feedback inhibition enhanced the impact of the donut-like motif (Fig. 2C, D: blue vs. purple, red vs. purple; *p* < 6.023 e-8 in both cases), without the donut-like motif, they were nearly ineffective, either individually or together, at signaling the strongest stimulus categorically (Fig. 2C, D: green, orange, brown). The magnitude of response variability (fano factor) in responses did not alter these findings (Fig. S1J).

Finally, we explored whether varying the values of various parameters in models not containing the donut-like motif, but containing feedback inhibition and/or recurrent amplification, might allow them as well to achieve categorical responses. We varied several key parameters (including slopes of the input-output functions), and simulated 324 circuit models representing different combinations of parameter values (Fig. S1K). We found that these models were still markedly less effective (at best, half as effective) at generating categorical responses compared to the model containing just the donut-like motif (Fig. S1L).

Donut-like inhibition, therefore, emerged as the most powerful single circuit motif (among the three proposed in the literature) for producing categorical neural responses (Fig. 2D).

### Functional pattern of competitive inhibition in the owl midbrain selection network is donut-like

To examine the potential role of donut-like inhibition in controlling categorical neural responses in the avian midbrain, we first investigated experimentally whether a donut-like inhibitory motif even operates in the owl midbrain selection network. As we pointed out above, anatomical tracing studies^31^ have indicated that the direct projections from Imc neurons to the OT spare the portion of the OT providing input to Imc, supporting a donut-like motif in the direct inhibitory pathway (Fig. 3A; white column – highlighting absence of purple projections in portion of OT containing black neuron), although this not been established functionally. Crucially, however, whether or not the indirect inhibitory pathway involving the Ipc also exhibits the donut-like motif is unknown (Fig. 3A, purple “?” under dashed projections and white column in Ipc). This is critical because the indirect pathway is known to be the dominant route of inhibition from Imc to OTid^31^: the majority of Imc projections target the Ipc^31^ (rather than the OTid), and Ipc provides substantial amplification of OTid responses to single stimuli^25^.

To determine whether the Imc-Ipc-OT circuit implements a functional donut-like pattern of competitive inhibition, we measured experimentally the strength of the net inhibition delivered by Imc neurons onto OTid neurons, due to both the direct as well as the indirect pathways. Specifically, we compared the strength of net inhibition from Imc neurons onto ‘misaligned’ OTid neurons encoding for stimuli at distant, non-matched azimuthal locations (“other”-inhibition; Fig. 3B), and separately, that onto “aligned” OTid neurons encoding for overlapping locations (“self”-inhibition; Fig. 3G). We did so by making dual extracellular recordings in the barn owl OTid and Imc (Methods; Fig. 3B, G), coupled with microiontophoretic silencing of Imc neurons^32^ (Methods).

To measure the strength of net “other”-inhibition, we first recorded the responses of OTid neurons to a stimulus (S1) inside the receptive field (RF; Fig. 3B, C-left; Methods) while simultaneously presenting a competing stimulus outside the RF (S2; at a distant azimuthal location from S1; Fig. 3D-left; Methods). We then repeated this measurement after focally (and reversibly) inactivating the portion of Imc encoding S2 (a site spatially mismatched with the OTid recording site; Fig. 3C-right, 3D-right), and compared the responses (Methods). Focal, space-specific Imc inactivation was achieved by iontophoresing the pan-glutamate receptor blocker, kynurenic acid^32^ (Methods).

Responses to S1 in the OTid are known to be divisively suppressed by a distant S2 (by an amount depending on their relative strength)^49,50^, and this suppression is known to be abolished upon focally inactivating the portion of Imc representing S2^32,33^. Therefore, any increase in OTid responses to the paired presentation of S1 and S2 following Imc inactivation would represent other-inhibition provided by Imc (Fig. 3D-right vs. 3D-left; Fig. 3E, red vs. black). We quantified the strength of this net other-inhibition as % change in OTid responses: 100*(responses in Imc intact condition − responses in Imc off condition) / responses in Imc off condition (Methods; no change in OTid responses would indicate zero competitive suppression by Imc onto that OTid neuron).

We found that Imc neurons exerted strong inhibition onto OTid neurons encoding for distant, non-matched spatial azimuths (Fig. 3F, L-red; mean strength = −40.47% +/− 17.70%, *n* = 19 paired neurons; *p* = 9.43e-9, *t* test against 0; mean distance between centers = 26.74°). We verified that the results were specifically due to Imc inactivation by observing that OTid responses to paired S1 and S2 returned to pre-drug levels after recovery from iontophoresis (measured 15 min after the drug was turned off; Fig. S2A; Methods). Indeed, the suppression provided by Imc accounted for nearly all the suppression exerted by the stimulus S2 (Fig. S2B). In these experiments, Imc was inactivated effectively (median = 95%, 95% CI of median = [87%, 103%]; *p* = 3.8e-6, sign test, *n* = 19; Fig. S2C).

Next, to measure the strength of net “self”-inhibition, we recorded the responses of OTid neurons to a single stimulus (S1) presented inside the RF (Fig. 3G, H-left; Methods). We then repeated this measurement after focally (and reversibly) inactivating the portion of Imc also encoding for S1 (a site spatially matched with the OTid recording site; Fig. 3H-right; Methods), and compared the responses.

Following a similar argument as above, any increase in OTid responses between the Imc-intact (Fig. 3I-left; 3J-black) and the Imc-inactivated (Fig. 3I-right; 3J-blue) conditions would represent self-inhibition provided by Imc. We quantified the strength of this net self-inhibition also as % change in OTid responses: 100* (responses in Imc intact condition − responses in Imc off condition) / responses in Imc off condition (Methods).

We found that Imc neurons exerted no significant inhibition onto OTid neurons encoding for overlapping spatial locations (Fig. 3K, L-blue; mean strength = −3.7%, s.d. = 12.2%, *n* = 28 neuron pairs; *p* = 0.12, *t* test against 0; mean distance between centers = 2.86 °). We verified that these results were not due to ineffectiveness of iontophoresis by observing that the suppression of Imc responses by kynurenic acid was substantial (Fig. S2C), and not distinguishable from that in the “other” case (Fig. S2C; *p* = 0.68, ranksum test, Imc suppression by drug in “self” vs. “other” cases). Therefore, strength of net self-inhibition in the OTid was substantially weaker than the strength of net other-inhibition (Fig. 3L, red vs. blue; *p* = 7.8e-11, two sample *t* test with HBMC correction; Methods). Additionally, the average strength of net self-inhibition from Imc to OTid was not significantly different from zero (Fig. 3L, blue; *p* = 0.12, *t* test with HBMC correction; Methods).

Together, these findings demonstrated the presence of a net functional donut-like pattern of competitive inhibition implemented by Imc across the OTid (azimuthal) space map; operating necessarily along both the direct and indirect pathways. Notably, they attest to the presence of a functional donut-hole of inhibition in Imc→OTid projections, as well as in Imc→ Ipc projections.

### Donut-like inhibition in the avian midbrain selection network is multi-holed

**Fig. 4:**
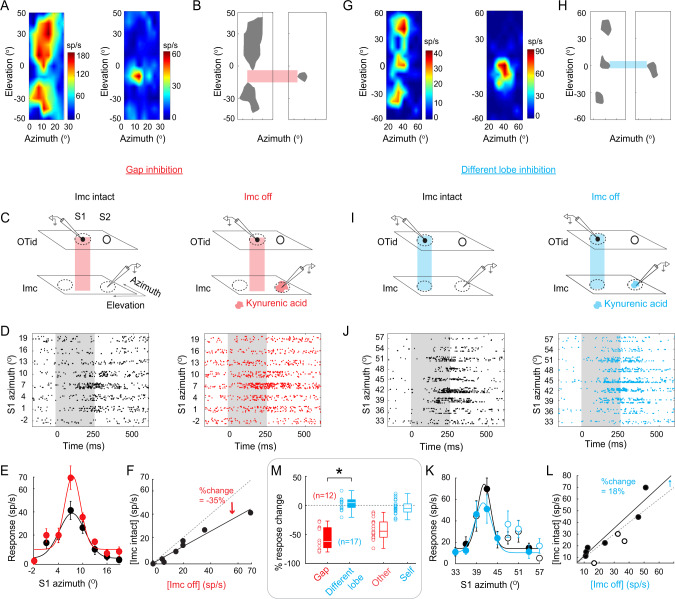
Barn owl midbrain selection network contains multi-holed donut-like inhibitory motif operating across 2-D sensory space (azimuth x elevation). **A**–**F** Measurement of the strength of net “gap” inhibition from Imc → OTid with paired recordings in owl Imc and OTid. **A** Spatial receptive fields (RFs) of an Imc neuron (left) and an OTid neuron (right) from a paired Imc-OTid recording experiment. Imc RF is two-lobed^51^(text; Fig. S3B–D; Methods). OTid RF lies in the gap between the lobes of the Imc RF. **B** Binarized versions of RFs in (**A**), at 60% max. firing rate in each case. Red horizontal bar: highlights the relative position of OTid RF to Imc RF lobes. **C**–**F** Conventions as in Fig. 3C–F. **C** Red vertical bar indicates that OTid RF is in the gap between Imc RF lobes. **E** mean ± s.e.m (*n* = 10). **F** Line: Best fit straight line; slope = 0.65, R^2^ = 0.95. % change in responses directly estimates the strength of inhibition at this OTid neuron due to Imc (here, −35%; Methods; conventions as in Fig. 3F). **G**–**L** Measurement of the strength of net “different lobe” inhibition from Imc → OTid with paired recordings in Imc and OTid. Conventions as in (**A**–**F**). **G**, **H** Three-lobed spatial RF of an Imc neuron (left; Fig. S3E–G; Methods). RF of OTid neuron overlaps (blue bar) with one of the lobes of Imc neuron’s RF. **K** mean ± s.e.m (*n* = 10). **L** Line: Best fit straight line; slope = 1.18, R^2^ = 0.9. % change in responses directly estimates the strength of inhibition at this OTid neuron due to Imc (here, 18%; Methods; conventions as in Fig. 3F). **M** Population summary of strength of net “gap” inhibition (red; *n* = 12), and strength of net “different lobe” inhibition (blue; *n* = 17) from Imc→ OTid. Open red and blue box plots: reproduced from Fig. 3L. *p* = 1.02e-6 (red vs. 0), *p* = 0.35 (blue vs. 0); *p < 0.05, *p* = 8.69e-11 (red vs. blue), paired two-sided *t* tests with HBMC correction. Box plot conventions as in Fig. 3L. See also Fig. S3. Source data are provided as a Source Data file.

Imc neurons are strikingly asymmetric in their encoding of elevational vs. azimuthal space. The majority (67%) exhibit RFs with multiple discrete firing fields (“lobes”) distributed along the elevation, but not the azimuth (multilobed RFs exhibit up to 3 lobes along the elevation^51^; Fig. 4A, G-left panels). This unusual RF structure has been shown to be essential for Imc to achieve selection at all possible pairs of spatial locations in the face of scarcity of its neurons, and it does so using a combinatorially optimized inhibitory strategy^51^.

A direct consequence of multilobed encoding of elevational space is that there are gaps between the lobes of an Imc RF, constituting locations that are outside that neuron’s RF (Fig. 4A, B-left panels; light red bar). If the donut-like inhibitory motif is to operate generally in the avian midbrain network to support selection along not only azimuthal locations, but also along elevational locations, then the spatial pattern of inhibition must respect the following strict conditions. A multilobe Imc neuron activated by a stimulus within one of its RF lobes must send strong competitive inhibition to OTid neurons encoding locations outside all of its RF lobes, and specifically, to neurons encoding locations in the gaps between RF lobes (strong “gap”-inhibition). By contrast, it should send weak or no inhibition to OTid neurons encoding locations within any of its RF lobes, and specifically, within its other RF lobes (weak “different-lobe” inhibition). Together, these predict a multi-holed donut-like pattern of net inhibition from Imc to OTid (Fig. S3A).

To test experimentally if this strict requirement holds true in the owl midbrain selection network, we again made dual extracellular recordings in the OTid and Imc, and measured directly the strength of gap-inhibition, and separately, the strength of different-lobe inhibition from Imc neurons onto OTid. We first recorded the responses of an Imc neuron, mapped out its spatial RF, and applied previously published analytical approaches to determine if it was a multilobed RF (two-lobed RF in Fig. 4A, B-left panels and Fig. S3B–D; three-lobed RF in Fig. 4G, H – left panels and Fig. S3E–G^51^; Methods). If so, we next measured the strength of gap inhibition by positioning a second electrode in the OTid such that the spatial RF of the OTid neuron was centered within the gap between Imc RF lobes (Fig. 4A, B-right panels and Fig. 4C-left, light red bar). We recorded the responses of the OTid neuron to a stimulus inside its RF (S1; Fig. 4C-left; Methods) while simultaneously presenting a competing stimulus (S2) at a distant location along the elevational axis such that S2 was within a lobe of the RF of the Imc neuron (Fig. 4C-left – S1 in the Imc RF gap denoted by light red bar, and S2 within an Imc RF lobe).

Alternatively, to measure the strength of different-lobe inhibition, we positioned the OTid electrode such that the spatial RF of the recorded OTid neuron overlapped one of the lobes of the Imc neuron’s RF (Fig. 4G, H-right panels and Fig. 4I-left, light blue bar). We recorded the responses of the OTid neuron to a stimulus inside its RF (S1; Fig. 4I-left; Methods) while simultaneously presenting a competing stimulus (S2) at a distant location along the elevational axis such that S2 was within a different lobe of the Imc neuron’s RF than S1 (Fig. 4I-left – S1 within one Imc RF lobe and S2 within different one – light blue bar).

In both cases, we compared OTid responses when Imc was intact (Fig. 4C, I-left panels) vs. when the portion of Imc encoding S2 was focally and reversibly inactivated using kynurenic acid iontophoresis (Fig. 4C, I-right panels). As before, any observed response increases directly estimated, respectively, the (net) strengths of gap-inhibition or different-lobe inhibition exerted by an Imc neuron onto the OTid space map (see also Methods, Paired OTid and Imc data collection, “Gap” inhibition section for explanation of why potential spread of kynurenic acid to also include Imc neurons encoding S1 does not present confounds to measurement of strength of gap inhibition).

We found that Imc neurons exerted strong gap-inhibition (Fig. 4D–F, M-red), but weak different-lobe inhibition onto OTid (Fig. 4J–L, M-blue). The mean strength of gap-inhibition (along elevation) was strong at −55.88% (Fig. 4M-red, s.d. = 20%, *n* = 12 neuron pairs; *p* = 1.02 e-6, *t* test against 0 with HBMC correction; Fig. S3I, J-red; Methods). The mean strength of different-lobe inhibition was very weak at 2.43% (Fig. 4M-blue, s.d. = 10.3%, *n* = 17 neuron pairs; *p* = 0.35, *t* test against 0 with HBMC correction; Fig. S3I, J-blue; Methods), and was not distinguishable from the average self-inhibition (Fig. 4M- blue vs. open blue; *p* = 0.09; two sample test with HMBC correction). Imc was inactivated effectively in both sets of experiments (median = 98.2%, 95% CI of median = [96.46–99.99%]; Fig. S3K).

Taken together, these results demonstrated that the Imc implements precisely organized, multi-holed donut-like patterns of net inhibition onto the OTid space map (azimuth and elevation), operating along both the direct and indirect pathways. Each Imc neuron’s net inhibitory action in the OTid creates a spatial pattern complementary to its (multilobed) RF structure (Fig. S3A).

### Donut-like inhibitory motif is necessary for categorization by avian midbrain selection network

**Fig. 5:**
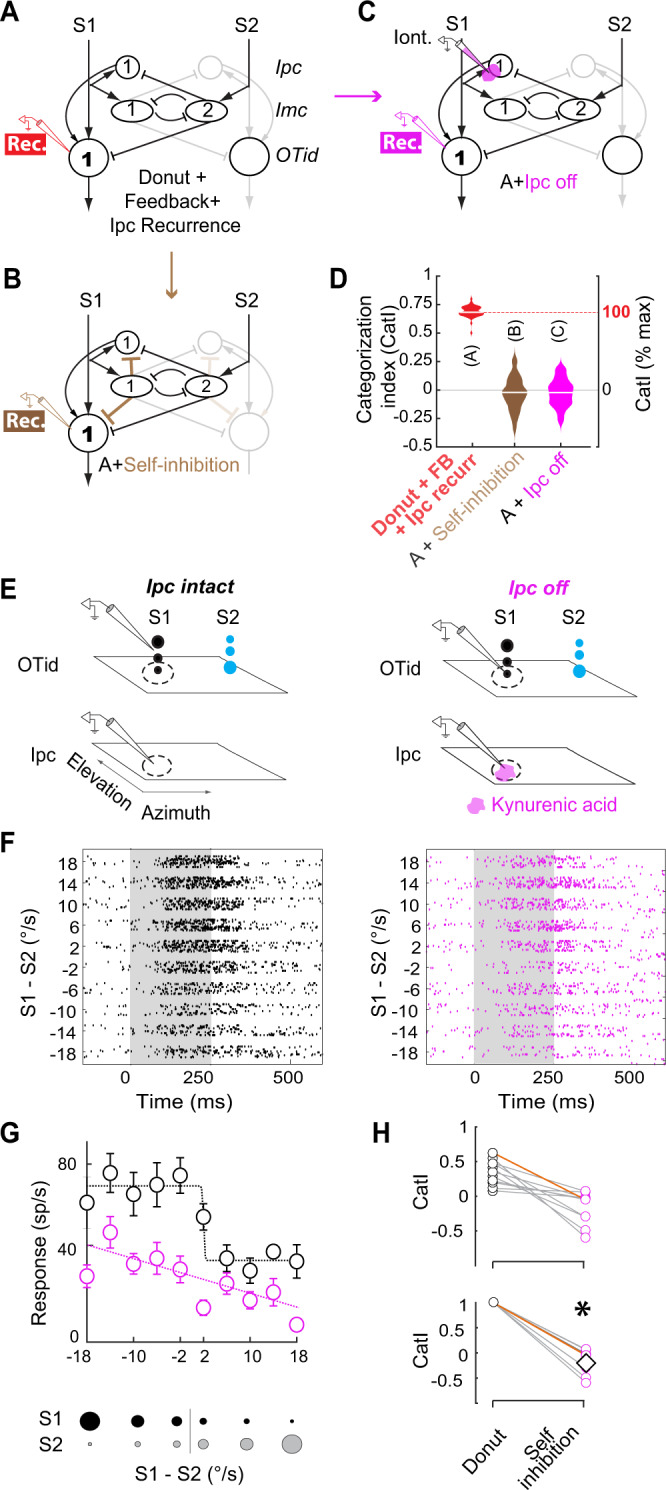
Donut-like inhibitory motif is required for categorical signaling of the strongest stimulus by the barn owl midbrain selection network. **A** Network model schematizing the OT-Imc-Ipc circuit with donut-like inhibition in the barn owl midbrain. “Rec”: Recording at OTid neuron 1. **B** Network model schematizing the desired experimental manipulation of disrupting donut-like inhibition (i.e., filling-in the donut-hole): introduction of self-inhibition (brown connections) in the direct pathway between Imc and OTid, as well as in the powerful indirect pathway through Ipc. However, this is infeasible experimentally (Figs. 3 and 4; text). **C** Network model schematizing the proposed equivalent experimental manipulation: focal inactivation of Ipc neuron in channel 1, mimicking the effect of introducing self-inhibition onto Ipc neuron 1, while recording from aligned OTid neuron 1. “Iont”: Iontophoresis of kynurenic acid (magenta blob) onto Ipc neuron; “Rec”: Recording OTid neuron. **D** Modeling results showing equivalence between desired (**B**) and proposed (**C**) manipulations. Plots of CatI computed from response profiles simulated from models in (**A**–**C**); conventions as in Fig. 2D; *n* = 50 model neurons; center lines in the violin plots indicate median values. Manipulations in both (**B**) and (**C**) cause abolishment of categorization. See also Fig. S4A–C showing, additionally, that (**C**) is not equivalent to removal of recurrent amplification. (Note: model in (**A**) is identical to model in Fig. 2A, right column-bottom; model in (**B**) is identical to model in 2B, middle column-third from top). **E** Paired recordings in OTid and Ipc such that RF of OTid neuron overlaps with that of Ipc neuron. Conventions as in Fig. 3C. Stimulus protocol used is the same two-stimulus morphing protocol used in model simulations in Fig. 2B–D; the relative strength between S1 and S2 was systematically varied. **F** Left: OTid response rasters in the Ipc-intact condition. Right: OTid response rasters in the Ipc-off condition. Distance between OTid and Ipc RF centers = 5°. **G** OTid response firing rates, computed from D over the 100–400 ms time window. mean ± s.e.m (*n* = 12). Black: Ipc-intact condition, magenta: Ipc-off condition. Dashed lines: best fitting sigmoid or straight line to data, chosen based on AIC criterion. Black: AIC (sigmoid) = 29.07, AIC (line) = 40.92; magenta: AIC (sigmoid) = 37.75, AIC (line) = 34.67. **H** Population summary of effect of Ipc inactivation on CatI; *n* = 11 neuron pairs; data in orange are from example neuron pair in (**F**, **G**). Top panel: measured values. Bottom panel: Data in top panel replotted after normalizing to Ipc-intact values. Diamond: average of magenta data; *p = 2.55e-5, two-sided ranksum test against 1 with HBMC correction. See also Fig. S4. Source data are provided as a Source Data file.

Considering the complexity of the multi-holed donut-like connectivity between Imc (and Ipc) and OT (Fig. 5A), we next asked if donut-like inhibition serves a functional purpose. Specifically, given its ability to produce categorical responses (modeling results; Fig. 2), we investigated whether this motif was necessary for the categorical signaling of the strongest stimulus by the OTid. To do  this, we needed to test the impact of causally disrupting the donut-like pattern of inhibition from an Imc neuron to OTid, on categorical signaling in the OTid. In other words, we needed to selectively introduce self-inhibition onto the spatially aligned OTid neuron, thereby “filling-in” the donut hole (Fig. 5B, brown projections). However, introducing self-inhibition experimentally by activating Imc → Ipc projections (indirect pathway) or Imc → OTid projections (direct pathway) between spatially aligned neuron-pairs (Fig. 5B, brown projections) is not feasible because such projections either do not exist or are functionally inactive (combined self-inhibition at most OTid neurons is weak or zero; Figs. 3L and 4M).

To resolve this apparent conundrum, we stepped back and considered the specialized anatomical connectivity of the avian midbrain selection network (Fig. 5A). We observed that in a competitive setting (i.e., when S1 and S2 are both present), the “amplifier” Ipc neurons corresponding to each stimulus are not free to amplify OTid activity solely based on that stimulus’s strength. For instance, whether Ipc neurons encoding for S1 amplify OTid responses to S1 at all, and if so, to what extent, are controlled by the strength of competitive inhibition due to S2 delivered by Imc neurons in S2’s channel (Fig. 5A, oval “Imc” neuron #2). In other words, in a competitive setting, the “amplifier” Ipc neurons operate wholly under the powerful control of the inhibitory Imc neurons.

This observation led us to reason that we could achieve the goal of experimentally introducing self-inhibition onto OTid neurons not by activating the (likely non-existent) self-projections from Imc to the aligned Ipc neuron in the dominant indirect pathway (Fig. 5B), but rather by mimicking the consequence of doing so, namely, by focally suppressing the evoked output of the aligned Ipc neuron (Fig. 5C, pink, drug iontophoresis onto aligned Ipc).

To test if this logic was sound, we first simulated this latter model (Fig. 5C), and found that focal Ipc-inactivation in the model abolished categorical signaling by OTid (Fig. 5D, pink vs. red), and this was indistinguishable from the effect of introducing self-inhibition (Fig. 5D, brown vs. red; simulation of 5B). This established the effectiveness of the proposed manipulation. Notably, this manipulation in the avian midbrain circuit during stimulus competition was akin to filling-in the donut hole, rather than to simply silencing recurrent amplification: simulating the silencing of just the recurrent amplification in a circuit model in which recurrence was not under the control of powerful competitive inhibition did not produce a significant drop in categorical signaling (Fig. S4A–C, blue vs. red-gray). In other words, the result of Ipc inactivation (5C, D) was nearly identical to that of introducing self-inhibition (5B, D), but very different from that of silencing recurrent amplification (Fig. S4B, C). These model simulations established that in the avian midbrain selection network, focal Ipc inactivation in a competitive setting (Fig. 5C) was functionally equivalent to the desired causal manipulation of filling-in the “hole” in the donut-like pattern of inhibition. Additionally, we confirmed that, as predicted by the specialized anatomical connectivity of the OT-Imc-Ipc network, silencing the Ipc neuron encoding for S2 (i.e., filling-in the donut-hole in channel “2”; Fig. 5C) had no impact on the competitive responses of OTid neuron encoding for S1 (i.e., in channel 1; Fig. S4D, E). This established that silencing just the Ipc neuron(s) encoding for S1 was sufficient to test the role of donut-like inhibition on categorical signaling by OTid neuron 1.

Using these insights, we proceeded to test experimentally, the functional consequence of filling-in the donut hole on categorization by barn owl OTid neurons. We focally (and reversibly) inactivated Ipc neurons by the iontophoresis of the pan-glutamate receptor blocker, kynurenic acid (Fig. 5E-right, pink blob, “Iont.”; Fig. S4F; Methods), while simultaneously recording the responses of spatially matched OTid neurons (“Rec.”). OTid responses were measured to the same two-stimulus morphing protocol used in our modeling (Fig. 5E left vs. right; protocol shown in Fig. 5G–bottom, same as Fig. 2C; Methods), as well as in previous experimental studies of categorical signaling in the OTid^8,22^.

We found that disrupting the donut-like inhibitory motif caused a substantial reduction in categorical signaling in the OTid (Fig. 5F, G: example neuron pair). Across the population of tested neuron pairs, categorization was nearly abolished by this experimental perturbation, with the median reduction in *CatI* of 104.7% (Fig. 5H; *n* = 11 neuron pairs; *CatI*: 95% CI of median = [91.52%, 117.92%]; *p* = 2.55 e-5, ranksum test against 1 with HBMC correction). These results reinforce the dominance of the indirect Imc-Ipc-OT pathway over the direct Imc-OT pathway (the donut-like inhibition there remained intact in these experiments). Thus, the midbrain spatial selection network not only contains a specialized donut-like inhibitory circuit motif, but also critically depends on it for categorical signaling by OTid.

## Discussion

Our results discover the donut-like inhibitory motif as a powerful, identifiable circuit mechanism for generating categorical neural selection boundaries, able to convert even linear response profiles to categorical ones (Fig. 2D-inset, 2D – orange/brown to purple; Fig. 5G, H – pink vs. black data).

### Superiority over feedback inhibition and recurrent excitation motifs for generating categorical neural responses

Contrary to prior proposals^42,44^, our results showed that feedback inhibition and recurrent amplification, by themselves, are not effective at producing categorical response profiles, nor is their combination (Fig. 2C, D). This was true independently of the strength of feedback inhibition (Fig. S1D), the strength of recurrent amplification (Fig. S1E), the specific implementation of recurrent amplification (Fig. S1F–H), the magnitude of response noise (Fig. S1J), and over a range of values of key parameters of the models including the slopes of the single-stimulus input-output functions (or neuronal “activation” functions; Fig. S1K, L). (The latter point is consistent with previous work showing that the steepness of input-output functions of neurons can be uncorrelated with whether the selection signal is categorical^9^). Although not effective for producing categorical responses, feedback inhibition and recurrent amplification have been linked to other important functions related to selection, namely, the implementation of a flexible selection boundary (feedback inhibition^13,44,52^), and evidence accumulation (recurrent amplification^39,42^). Selection circuits may, therefore, need to include these motifs for other reasons than categorization. If present as well, they heighten the efficacy of the donut-like inhibitory motif in producing categorical response profiles (Figs. 2D and S1H), effectively helping implement attractor dynamics for flexible and categorical decision-making^2,40,44^.

A viable alternative to donut-like inhibition for categorization are highly recurrent, non-structured networks, which have been shown in modeling to be capable of generating categorical outputs from a multiplexed representation of inputs^53,54^. However, because it is difficult to extract specific, experimentally testable neural circuit mechanisms from the opaque connectivity diagrams of recurrent networks, and in light of the recently reported counterpoint to mixed-selectivity descriptions^10^, we focused, here, on structured circuit mechanisms, and identified donut-like inhibition as being highly effective for categorizing inputs.

### Superiority over the normalization model for generating categorical neural responses

**Fig. 6:**
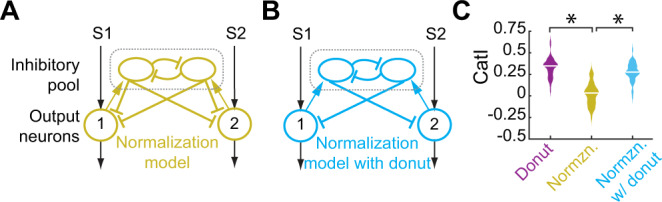
Divisive normalization model is not effective at producing categorical response profiles. **A** Schematic of normalization circuit model with pooled inhibition and inhibitory feedback^55,56^. **B** Schematic of normalization circuit with self-inhibition removed (i.e., with donut-like inhibition introduced). **C** Plot of CatI computed from the responses of neuron 1 in the normalization model (**A**; gold data) and in the model lacking self-inhibition (**B**; blue data), to the standard two-stimulus morphing protocol (as in Fig. 2C); *n* = 50 model neurons; center lines in the violin plots indicate median values. For comparison, the CatI values for the model from Fig. 2D that contains the donut-like motif (purple), is reproduced here; *p* = 5.2e-23 (gold vs. purple), *p* = 8.8e-19 (gold vs. blue), paired two-sided *t* tests with HBMC correction. Source data are provided as a Source Data file.

The structured donut-like organization of inhibition stands in contrast to another computational mechanism that has been invoked in the decision-making literature, namely divisive normalization^46,55–57^. This involves inhibitory elements that pool the drive from all the active channels (as opposed to receiving selective drive), that inhibit one other, and that deliver pooled inhibition uniformly (rather than in a donut-like manner) to the output elements (Fig. 6A^55,56^). The midbrain spatial selection network (OT-Imc-Ipc), in which categorical neural responses have been reported, does not implement pooled divisive normalization for selection across space: past work has shown that the inhibitory Imc neurons receive selective input^31–33,51^, and our results (Figs. 3 and 4) show that pattern of inhibitory output across the OT is not uniform (but rather donut-like).

To test more generally whether the divisive normalization model is capable of generating categorical response profiles, we simulated a circuit model of normalization and obtained model neuron responses to the two-stimulus morphing protocol (Fig. 6A). This model yielded a substantially lower *CatI* than the donut-like inhibitory motif (Fig. 6C right panel: mean *CatI* = −0.05 for normalization model compared to 0.331 for donut model in Fig. 2A, *p* = 1.5 e-29, *t* test with HMBC correction, gold vs. purple; left panel: *p* = 1e-12, *t* test of gold vs. purple). Thus, the normalization mechanism is not effective for generating categorical responses (consistent with findings from modeling in visual cortex^58^). Indeed, it was the presence of self-inhibition, specifically, that caused this circuit to be ineffective: a modified version of the circuit which did not include self-inhibition (Fig. 6B), did produce categorical responses (Fig. 6C, blue vs. gold data), further attesting to the primacy of the donut-like motif for categorization.

### Alternate implementation of the donut-like inhibitory motif

**Fig. 7:**
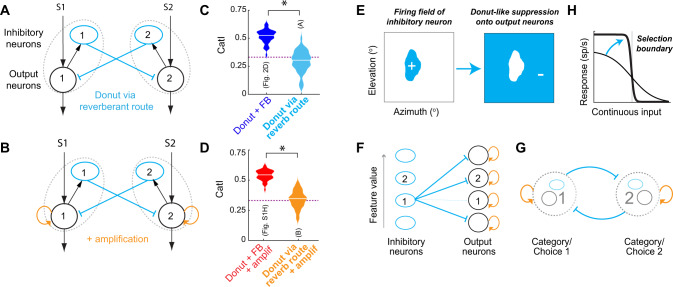
Alternate implementation (and generality) of the donut-like inhibitory motif. **A**, **B** Circuit with implementation of donut-like motif purely via a reverberant route, i.e., in the absence of any feedforward inhibition. **A** Without recurrent amplification. **B** With recurrent amplification; curved orange arrow. Blue ovals: inhibitory neurons, black circles: excitatory/output neurons, dashed gray ovals: populations of neurons representing each stimulus or category. **C**, **D** Plots of CatI computed from the responses of output neuron 1 in circuits in (**A**) (**C**, filled light blue data) and in (**B**) (**D**, filled orange data), to the standard two-stimulus morphing protocol (as in Fig. 2B). For comparison, the CatI values for the corresponding models with feedforward implementation of the donut-like motif are reproduced here (in **C**: dark blue data from Fig. 2D; in **D**: red data from Fig. SH). *p < 0.05. Filled light blue vs. dark blue, *p* = 1.7e-25; orange vs. red, *p* = 2.5e-31; paired two-sided *t* tests with HBMC correction. Purple dashed line: CatI of purple model from Fig. 2A, which has just the feedforward donut-like motif sans feedback; reproduced here from Fig. 2D. *n* = 50 model neurons; center lines in the violin plots indicate median values. **E**–**H** Graphical summary of central findings of this study. **E** Donut-like inhibition, i.e., inhibition (−) driven by preferred inputs (+) and delivered to all non-preferred inputs, can be implemented in neural circuits either in a feedforward manner (**F**) or in a reverberant manner (**G**), to generate categorical selection boundaries (**H**). **F**, **G** Blue ovals: inhibitory neurons, black circles: excitatory/output neurons. **F** dashed thin blue arrow indicates absence of inhibitory projection. **G** based on Figs. 1–4; (**G**) is a simplified representation of model in (**B**), in which populations of neurons representing each category or choice (dashed gray circle) mutually inhibit one another. The similarity of this reduced model structure to several previous models of selection highlights the generality of our findings and provides a mechanistic explanation for the ability of those models to produce categorical selection boundaries^40, 64, 67, 68^; see text. **H** Ability of donut-like motif to convert linear response profiles (thin black) to categorical (thick black) ones. Source data are provided as a Source Data file.

Both in the barn owl brain and in the various models considered in Figs. 2–5, the circuit architectures included feedforward inhibition from inhibitory (“Imc”) neurons to the output (“OTid”) neurons, with the donut-like motif instantiated as the absence of feedforward self-inhibition. However, other established models of decision-making do not include feedforward inhibition, but rather only involve inhibition in a ‘reverberant’ path between the competing options^40^ (Fig. 7A). To test if the implementation method impacted our conclusions, we simulated a version of our circuit model that implemented the donut-like motif only via a reverberant route (Fig. 7A); we note that this implementation, by definition, also includes feedback inhibition between the two channels (Fig. 7A: black neuron 1→ blue neuron 1→ black neuron 2 → blue neuron 2 → black neuron1). For completeness, we simulated this model both without (Fig. 7A) and with recurrent amplification within each channel (Fig. 7B; curved orange arrow). We found that this alternate implementation of the donut-like motif also successfully produced categorical response profiles (Fig. 7C, D; *CatI* = 0.29 (filled light blue), 0.34 (filled orange)). Nonetheless, the feedforward implementation of the donut-like motif (Fig. 2A, B, right panels) offered additional benefits to categorization beyond the purely reverberant implementation in each case (Fig. 7C: filled light blue vs. dark blue; 7D: filled orange vs. red).

### Generality of donut-like inhibition as mechanism for categorical neural responses

This study revealed the donut-like inhibitory motif as the engine of categorization in the avian midbrain network for spatial selection, a network conserved across vertebrates^21,31,59,60^. Although the response curves discussed here were obtained using competing looming stimuli, extensive work in the barn owl has shown that categorical response profiles in the owl midbrain are not specific to looming visual stimuli. Rather, they occur no matter what the varying stimulus feature is (stimulus contrast, for instance), and no matter what the sensory modality is (for instance, binaural level of an auditory stimulus)^9,22,28,61^. In other words, categorical neural responses are not idiosyncratic to a specific stimulus feature, but are in fact, general, representing the neural basis of stimulus competition and selection across space in the barn owl^14,21^. Notably, the categorical responses measured in the owl OTid have been shown to be capable of accounting for behavioral deficits in target selection for spatial attention in monkeys following focal perturbation of the homologous SCid^14^. These observations, together with the fact that the midbrain tecto-isthmic selection network is conserved across all vertebrates^26,31,59,60,62,63^, point to the potential generality of the donut-like motif across the vertebrate midbrain for spatial selection.

Might this motif also generalize to other forms of categorical selection (beyond selection across space), mediated by other cortical and subcortical areas (beyond the midbrain)? If so, how might it generalize, implementation-wise? Below, we examine these two questions in order.

To address the first question of whether or not this motif might generalize (issue of feasibility), we draw upon the extensive literature of computational modeling of categorical decision-making by various cortical (and subcortical) areas^2,5–7,11,40,64–67^. These models successfully account for neural as well as behavioral responses in a wide array of decision-making tasks –perceptual decision-making, delayed-match-to-sample decision-making, sensory categorization, etc. Examination of the circuit architectures in these models reveals that nearly all of them contain a (seemingly hidden) donut-like inhibitory motif^34^. Some include a feedforward implementation^64,65,67^ (as in Figs. 2 and  S1), while others include a reverberant implementation (as in Fig. 7B)^40,44,64,67,68^. The existence of this motif in these models, however, is not discussed explicitly, and the need for it is unclear. We posit, based on our experimental as well as modeling findings here, that it is this motif that imbues those models with the ability to categorize. Indeed, the one class of models that does not contain the donut-like motif is a normalization-based modeling of decision-making^69–73^, which we show is ineffective for generating categorical neural response (Fig. 6). Together, these observations support the feasibility of the donut-like motif for producing categorical neural representations across brain areas, animal species and task contexts.

To address the second question of how it might generalize (issues of implementation), we highlight four key characteristics of the donut-like motif in the avian midbrain selection network and discuss plausibly generalizable implementations of each. First, in the midbrain selection network in which the donut-like motif operates, individual stimuli are encoded with neural activity that is proportional to their net priority^21–23^. This readily generalizes to other instances of selection in which stimulus options are encoded with neural activity that is proportional to their net attractiveness or importance: for instance, subjective-value of an option in the case of value-based decision-making^74–76^, degree of membership of a stimulus in the case of perceptual categorization^2,77^, etc. Second, in this network, long-range suppression across spatial locations is the substrate upon which the donut-like inhibitory motif is sculpted, and is implemented by inhibitory neurons with far-reaching projections across the space of stimuli. Alternatively, such inhibition could also be implemented, for instance, in cortical circuits, through long-range excitation contacting local inhibitory neurons. Third, in the midbrain network, the donut-like motif is instantiated via a feedforward implementation. As we saw in Fig. 7A–D, an equivalent reverberant implementation, found in many models of selection^40,78^ is also effective. Fourth, in the midbrain network, the donut-like motif aids spatial selection by operating across a well-organized topographic map of space. However, for many forms of selection, such organized functional maps do not exist, with olfactory categorization being an extreme example^2,79^. In the case of olfactory decision-making, there is some evidence suggesting self-sparing, combinatorial lateral inhibition across mouse glomeruli^79,80^, of the kind found in the owl Imc. In most of these cases, however, a detailed description of the large-scale connectivity diagrams of inhibitory neurons in the corresponding brain areas is yet to be worked out; this study suggests that a search for donut-like inhibitory connectivity might be a fruitful endeavor.

More generally, the donut-like motif described here does not rely on mechanisms of plasticity for its operation. It is able to dynamically generate categorical neural responses from continuously varying inputs, on the fly, and dynamic categorization is critical to various behaviors (value-based decision-making, action selection, spatial attention, etc.). However, conceptually, this motif could also be introduced into a circuit through plasticity mechanisms, thereby aiding in the generation of categorical response profiles that are learned with experience^6,12,81,82^.

### Temporal aspects of selection, and links to behavior

In this study, we were interested, particularly, in mechanisms that could generate categorical profiles of average firing rates (steady state neural activity) as a function of relative stimulus strength, as reported experimentally in the barn owl midbrain^9,22,28,61^. (Similar categorical firing rate profiles have also been reported in multiple other brain areas across species and task configurations^2,5–13^). Our past modeling work (which forms the basis of this work) has shown that not including precise timing descriptions—for instance, timing of the arrival of excitation vs. inhibition, does not impact the ability of these models to capture phenomena at the level of average firing rates^22,44^. Separately, several experimental studies have also reported temporal aspects of decision-making—time courses of neural activity as well as behavioral reaction times. We recall that leading classes of models of selection/decision-making contain the donut-like inhibitory motif^2,5–7,11,34,40,64–67,83^. Consequently, because those models account for experimentally measured reaction times of animals during the selection tasks (as well as in some cases, response time courses), the donut-like motif is consistent with such temporal aspects and dynamical features of categorical decision-making as well. It will be valuable for future studies (involving behavior) to explore these links explicitly.

The central proposal of this study is that donut-like inhibition underlies categorical neural responses. Whereas neural response profiles underlying decision-making and selection tasks are frequently categorical^2,5–13^, psychometric profiles (of accuracy) are often not. To clarify, whereas the animal or subject must (and does) make a discrete choice on each trial, which results in the task being referred to commonly as a “categorical” decision-making task, behavioral performance, when assessed as a function of a continuous task parameter, is often not actually step-like^17,76,84,85^. It does not exhibit a large, abrupt change across the category boundary, but instead, varies more gradually across it. These two facts, however, are not in conflict: in line with previous findings, neurons can encode information more effectively than the animal as a whole, with behavior being a result of (noisy) aggregation of activity across neurons^86^. Consequently, the central prediction of this study is that causally perturbing donut-like neural inhibition in a relevant brain area during a decision-making task, will cause loss of categorical neural responses, and in turn cause psychometric response curves to become shallower (more gradual) than in the intact condition, independently of their original shape, with selection performance worsening particularly around the selection boundary.

In closing, we propose that the donut-like inhibitory motif (Fig. 7E–H) may be a critical neural circuit module common to various forms of categorical selection and decision-making. An intriguing open question in this context, even in the avian midbrain selection network, is how the wiring of the exquisitely organized donut-like connectivity is achieved in the brain.

## Methods

### Animals

We performed experimental recordings in 7 head-fixed awake adult barn owls viewing a visual screen passively (*Tyto alba*). Both male and female birds were used; the birds were shared across studies. All procedures for animal care and use were carried out following approval by the Johns Hopkins University Institutional Animal Care and Use Committee, and in accordance with NIH guidelines for the care and use of laboratory animals. Owls were group housed in flight runs within the aviary, each containing up to 6 birds. The light/dark cycle was 12 hr/12 hr.

### Neurophysiology

Experiments were performed following protocols that have been described previously^32,49^. Briefly, epoxy-coated, high impedance, tungsten microelectrodes (A-M Systems, 250 μm, 5–10 MΩ at 1 kHz) were used to record single and multi-units extracellularly in the OTid. Multi-barrel glass electrodes (Kation Scientific, Carbostar– 3LT, 0.4-1.2MΩ at 1 kHz) filled with kynurenic acid (a competitive inhibitor of ionotropic glutamate receptors; pH 8.5–9 at a concentration of 40 mM) were used to record from and inactivate neurons in the Imc and Ipc. Inactivation was performed using micro iontophoresis by ejecting kynurenic acid with an eject current of −450 nA to −500 nA; data were collected starting 15 min after drug ejection commenced. A retain current of +15 nA was used to prevent leakage of the drug from the tip of the electrodes when drug was not being iontophoresed. Recovery data were measured 15 min after drug ejection was ceased. Microiontophoresis, the technique we use here, is an established technique for focal delivery of drugs that has been used extensively for this purpose in the literature^87–91^. Prior published work has demonstrated that microiontophoresis of kynurenic acid is spatially specific in the Imc-OT circuit^32^, and that it offers a reliable approach to quantify Imc inhibition onto OTid neurons^32^. Additionally, it is preferable to the alternative of electrical microstimulation of Imc neurons for measuring the strength of Imc-OT inhibition because, (a) it avoids the confound that can arise from potentially activating fibers of passage from Ipc to OT as well (which are known to course through the Imc region^26,31^), and (b) it measures the strength of physiological inhibition evoked by a stimulus rather than that evoked by ectopic electrical activation.

### OT, Imc and Ipc targeting

We navigated to the OT (based on well-established methods^92^), and then navigated to the Imc using the OT’s topographic space map as reference. The Imc is an oblong structure that is 2.8 mm rostrocaudally and 0.35 mm dorsoventrally, appearing as a 700-μm × 350-μm elliptical disk in coronal sections. It lies parallel to the rostrocaudal axis of the OT, located ~16 mm ventral to the surface of the brain, and ~500 μm medial to the medial-most part of the OT. We targeted the Imc following published methods^32^. Imc targeting has been validated previously using dye injections and lesions^51^. Dorsoventral penetrations through the Imc were made at a medial-leading angle of 5˚ from the vertical to avoid a major blood vessel in the path to the Imc. The Ipc lies roughly 500–700 um medial to the Imc and its targeting was confirmed based on the neural response characteristics of the neurons (characteristic bursty responses^61^).

### Data collection and spike sorting

Multi-unit spike waveforms were recorded using Tucker Davis Technologies hardware interfaced with MATLAB. The responses of neurons were measured by counting spikes during a time window (typically 100–350 ms) following stimulus onset.

The automated “wave-clus” spike-sorting toolbox was used for spike sorting^93^. We included only those units for analysis for which fewer than 5% of the recorded spikes were within 1.5 ms (inter-spike interval; ISI) of each other.

Model details (Related to Figs. 2, S1, S1, 6 and 7).

### Input output functions

The input output functions (firing rate $f$, as a function of the saliency, $l$) of the neurons in the model were simulated using sigmoid functions using previously published methods^44^.

1 $$f\left(l\right)=c+s\left(\frac{{l}^{m}}{{l}^{m}+{L}_{50}^{m}}\right)$$

where $c,$ is the baseline firing rate of the neuron; $l$, is the saliency parameter of the stimulus (e.g., loom speed of the stimulus, loudness of an auditory stimulus, contrast of the stimulus, speed of a moving stimulus) that can vary continuously over a range; $s,$ is the maximum change in the firing rate of the neuron; ${L}_{50}$ is the saliency value at which the neuron’s firing rate changes by 50% of the maximum change; and m, is a parameter that controls the slope of the sigmoid.

### Excitatory neurons

The excitatory neurons were simulated using the following parameters (which are consistent with the parameters obtained by fitting a sigmoid to response functions of OTid neurons^44^):

2 $$c=5.3,s=22.2,{L}_{50}=11.6,m=2$$

### Inhibitory neurons

The inhibitory neurons were simulated using the following parameters (which are consistent with the parameters obtained by fitting a sigmoid to response functions of Imc neurons^44^):

3 $$c=5,s=15,{L}_{50}=8,m=10$$

### Recurrent excitation neurons

The excitatory neurons that provide recurrent amplification in Fig. 2 were simulated using the following parameters (which are consistent with the parameters obtained by fitting a sigmoid to response functions of Ipc neurons^48^):

4 $$c=8.4,s=36,{L}_{50}=5.8,m=3.3$$

The inhibition sent from the inhibitory neurons onto excitatory neurons is modeled using input and output divisive factors as below using previously published methods^44^.

5 $$f=\left(\frac{1}{{s}_{{out}}}+1\right).\left(\frac{c}{{s}_{{in}}+1}+s\left(\frac{{l}^{m}}{{l}^{m}+{L}_{50}^{m}+{s}_{{in}}^{m}}\right)\right)$$

where

- ${s}_{{in}}={d}_{{in}}.I,{s}_{{out}}={d}_{{out}}.I$ are the input and output divisive factors.
- *d*_*in*_ and *d*_*out*_ are parameters that control the strength of input and output division and $I$ is the output (firing rate) of the inhibitory neuron sending the inhibition.

The value of these parameters chosen were *d*_*in*_ = 0 *and d*_*out*_ = 0.25 consistent with previously published methods (see Fig. 5D in^44^).

### Feedback inhibition

In models in which the inhibitory neurons inhibit each other (Fig. 2A, middle column-top; green inhibitory connections), the feedback inhibition was modeled as below using previously published methods^44^.

6 $$I(t)=\left(\frac{1}{{i}_{{out}}}+1\right).\left(\frac{c}{{i}_{{in}}+1}+s\left(\frac{{l}^{m}}{{l}^{m}+{L}_{50}^{m}+{i}_{in}^{m}}\right)\right)$$

7 $${i}_{{in}}(t)={r}_{{in}}.I^{\prime} (t-1),{i}_{{out}}={r}_{{out}}.I^{\prime} (t-1)$$

where $I^{\prime}$ is the output (firing rate) of the other inhibitory neuron at time $(t-1)$.

These equations were iteratively applied until there was no further change in the output of the neurons (i.e., steady state was reached). The time course of reverberant activity due to this feedback connectivity has been plotted in our previously published work^44^.

The values of the feedback parameters used were ${r}_{{in}}=0.8,{r}_{{out}}=0.01$ consistent with previously published methods. We also varied these two parameters (varying feedback; Fig. S1D) to study their effect on categorization index.

### “Self” inhibition and donut

In models in which the excitatory neuron receives inhibition from more than one inhibitory neuron (e.g., Fig. 2A, left column top model; baseline model), the inhibition from these sources was combined as below.

8 $$f=\left(\frac{1}{{s}_{{out}1}}+1\right).\left(\frac{1}{{s}_{{ou}t2}}+1\right).\left(\frac{c}{{s}_{{in}1}+{s}_{{in}2}+1}+s\left(\frac{{l}^{m}}{{l}^{m}+{L}_{50}^{m}+{s}_{{in}1}^{m}+{s}_{{in}2}^{m}}\right)\right)$$

where, $${s}_{{in}1}={d}_{{in}}.{I}_{1},{s}_{{out}1}={d}_{{out}}.{I}_{1},{s}_{{in}2}=({s* d}_{{in}}).{I}_{2},{s}_{{out}2}=({s* d}_{{out}}).{I}_{2}$$

${I}_{1}$ is the output (firing rate) of the inhibitory neuron 1 and ${I}_{2}$ is the output (firing rate) of the inhibitory neuron 2.

The parameter value $s$ controls the strength of self-inhibition, and ranges between 0 and 1. In models that have “donut-like connectivity”, *s* is set to 0 (e.g., Fig. 2A, middle column-second from top model; absence of purple projections from Imc neurons to aligned OTid neurons; Donut model). In models which do not have the donut-like connectivity (e.g., Fig. 2A, left column top model; baseline model), $s$ is set to 1.

We also vary the value of $s$ systematically between 0 (donut) and 1 (maximum self-inhibition) to study the effect of the strength of “self” inhibition on categorization index (Fig. S1I).

### Recurrent excitation

In the models with recurrent excitation, (of the kind in Fig. S1F), the output of the neuron is scaled by a factor (k; k = 2.5 in Fig. S1G, H).

In the model in Fig. 2, the output of the neuron which receives recurrent amplification is modeled as below.

9 $$f(l)=\left(1+{e}_{{out}}\right).\left(\frac{1}{{s}_{{out}}+1}\right).\left(\frac{c}{{s}_{{in}}+1}+s\left(\frac{{l}^{m}}{{l}^{m}+{L}_{50}^{m}+{s}_{{in}}^{m}}\right)\right)$$

where, ${e}_{{out}}={e}_{a}.A,{e}_{a}=0.01$, and $A$ is the output of the neuron sending the amplification.

The value of the parameter is chosen as 0.01 to yield results consistent with the amplification effect of Ipc on OT responses as reported in previously published work (Ipc inactivation results in a 31% decrease in the OTid responses on average^25^). We also vary the amplification factor ${e}_{a}$ to study its effect on categorization index (Fig. S1E).

### Models for Figs. 6 and 7

To implement the models in Figs. 6 and 7 (normalization model and donut-like motifs purely via a recurrent route), we used the input-output functions and the effect of divisive inhibition described above. The primary feature of these models that is different from models in Figs. 2, and S1 is that the excitatory neurons send inputs to inhibitory neurons, which then inhibit those excitatory neurons. The output of an excitatory neuron at time t is calculated by applying divisive inhibition (from the inhibitory neurons at time t − 1) to its activity at time t − 1. This activity is then used to calculate the activity of the inhibitory neurons at time t as below.

10 $$I(t)=\left(1+{e}_{{out}}\right).I(t-1)$$

where, ${e}_{{out}}={e}_{a}.A,{e}_{a}=0.01$, and $A$ is the output of the neuron sending the input at time t − 1.

This process is repeated iteratively until steady state activity is obtained. The initial activities (at t = 0) of the inhibitory neurons are set to their baseline level, and of the excitatory neurons are calculated from the stimulus inputs without any divisive inhibition.

For the models with recurrent amplification (Fig. 7A, B), the output of the neuron is scaled by a factor (k, k = 2.5).

#### Calculating categorization index (Figs. 2, 6, 7, S1 and S4) and boundary discriminability (Fig. S1) for the models

For each of the models in Figs. 2, 6, 7, S1 and S4 we used a two-stimulus strength morphing protocol described in previously published work^8^. We presented stimulus S1 at location 1 and stimulus S2 at location 2. As the strength of the first stimulus was increased, the strength of the second stimulus was decreased (Figs. 2B and 5E). Responses of the model output neuron (#1) were simulated using this protocol. Random noise from a standard normal distribution was added to the responses. The variance of the noise added depended on the mean value of the responses (m) and a fano factor value (ff): variance = ff*m. This was repeated 30 times (to mimic 30 reps of data collection from a ‘neuron’) and was used to compute the response profiles (mean +/− s.e.m) of that neuron from different model circuits. Similarly, response profiles for 50 neurons were obtained for each model circuit. For all the model runs reported, we used a fano factor value of 6. We also varied the fano factor value to test the effect of noise on model performance (Fig. S1J).

This stimulus presentation protocol results in 2 categories (Category 1: S1 > S2 and Category 2: S1 < S2). We measured the boundary discriminability (bd’) of the responses of neuron 1 by calculating the d-prime between the responses of neuron to stimuli pair straddling either side of the selection boundary and at a distance of 3 units from the boundary as:

11 $${{bd}}^{{\prime} }=\frac{{{{{{{\rm{\mu }}}}}}}_{1}-{{{{{{\rm{\mu }}}}}}}_{2}}{\sqrt{0.5\left({s}_{1}^{2}+{s}_{2}^{2}\right)}}$$

where, ${{{{{{\rm{\mu }}}}}}}_{1}$ and ${{{{{{\rm{s}}}}}}}_{1}$are the mean and the standard deviation of the responses of the neuron to the stimuli pair from category 1 near the boundary; and ${{{{{{\rm{\mu }}}}}}}_{2}$ and ${{{{{{\rm{s}}}}}}}_{2}$are the mean and the standard deviation of the responses of the neuron to the stimuli pair from category 2 near the boundary.

To compute the categorization index, we compared two quantities: (modified from previously published work^6,8^) (a) the mean within-category d-prime (WCD’) between the responses of the neuron to pairs of stimulus-pairs (S1 and S2) that are in the same category, and (b) the mean between-category d-prime (BCD’) between the responses of the neuron to pairs of stimulus-pairs that are in different categories. The pairs are chosen while ensuring that (i) the number of pairs used to calculate both these metrics are the same, and (ii) the distribution of the distances between the chosen pairs for calculating both the metrics are the same. The categorization index is calculated from these two metrics as:

12 $${{{{{\rm{Categorizationindex}}}}}}\,({{{{{\rm{CatI}}}}}})=\frac{{{{{{\rm{mean}}}}}}({{{{{\rm{BCD}}}}}}^{\prime} )-{{{{{\rm{mean}}}}}}({{{{{\rm{WCD}}}}}}^{\prime} )}{{{{{{\rm{mean}}}}}}({{{{{\rm{BCD}}}}}}^{\prime} )+{{{{{\rm{mean}}}}}}({{{{{\rm{WCD}}}}}}^{\prime} )}$$

CatI = 1 indicates idealized, step-like responses; =0 indicates linear, non-categorical responses; <0 indicates better discriminability within category than between categories (Fig. S1C).

### Data collection protocol

Visual stimuli used here have been described previously^8,49^. Briefly, looming visual dots are flashed at different locations on a tangent TV monitor in front of the owl. Looming stimuli were dots that expanded linearly in size over time, starting from a size of 0.6° in radius. The speed of the loom was decided based on the stimulus protocol and varied between 9.6°/s and 21.6°/s. Visual stimuli were presented for a duration of 250 ms with an inter stimulus interval of 1.25 s to 4 s.

### Receptive fields (RFs)

For measuring spatial RFs of Imc, OTid and Ipc neurons, a single stimulus of a fixed contrast was presented at the sampled locations. The order of locations at which the stimulus was presented was randomized to minimize adaptation. A location is considered to be inside the RF if it evokes responses significantly different from that of baseline response. All other locations are considered to be outside the RF.

The following stimulus protocols were used for measuring data reported in Figs. 3 and 4.

I. Paired OTid and Imc data collection

1. “Other” inhibition

To measure the strength of “other” inhibition sent from an Imc neuron to a distant location in the OTid space map, we simultaneously recorded from the Imc neuron (with a multi-barrel glass electrode filled with the kynurenic acid), and a spatially misaligned “other” OTid neuron (with a tungsten electrode) as described below; we ensured that the half-max of the RF of the OTid neuron lay outside (did not overlap with) the half-max of the Imc RF.

For measuring “other” inhibition, we recorded the following data curves together in an interleaved manner.

- Tuning curve (TC) centered at the OTid RF peak: A 1-dimensional spatial tuning curve centered at the peak of the OTid RF. The stimulus had a loom strength of 9.6°/s.
- Tuning curve centered at the OTid RF peak + competing stimulus centered at the Imc RF peak (TCC): The same curve as in a), but along with a (distant) competitor positioned at the peak of the Imc RF. The strength of the competing stimulus was chosen to be 19.2°/s. This second competing stimulus drives the Imc neuron, which sends strong competitive inhibition to the OTid neuron. Since the competing stimulus was more salient than the stimulus driving the OTid neuron, the responses of the OTid neuron were strongly suppressed consistent with previous published results^49^.
- Tuning curve (TC) centered at the Imc RF peak: 1-dimensional spatial tuning curve centered at the Imc RF. The loom strength of the stimulus was chosen to be 9.6°/s.

We measured the above 3 curves both when the Imc neuron is intact (“baseline” condition) and focally inactivated (“inactivation” condition) and compare the responses as below. In a subset of the data, we also measured the curves after the responses of the Imc neurons recovered (Fig. S2A, “recovery” condition) from focal iontophoretic inactivation. Inactivation curves were measured 15 min after the drug ejection was started. Recovery curves were measured 15 min after the drug ejection was stopped.

We analyzed the data from these three curves as below.

First, a distant competing stimulus is known to typically suppress OTid responses^49,50^, something that we confirmed by comparing the responses of OTid neurons to the TC (curve a) and TCC (curve b); Fig. S2B. Notably, it is also known that some OTid neurons do not show such suppression, and any such neurons were excluded from our analyses^9^.

Next, to measure the amount of Imc inactivation, we compared the responses of the Imc neurons to the TC measured at the Imc RF peak (curve c) in the baseline condition vs. the inactivation condition. Kynurenic acid was able to effectively shut down Imc responses: (Fig. S2C; red, median strength of Imc inactivation = 95%, 95% CI of median = [87%, 103%], *p* = 3.8e-6, sign test, *n* = 19).

Finally, to measure the strength of “other” inhibition, we compared the TCC responses (curve b) in the baseline condition and the inactivation condition. In the baseline condition, the Imc neuron was intact, driven by the competing stimulus and sent strong inhibition to the OTid neuron. In the inactivation condition, the Imc neuron was silenced, as a result of which the OTid neuron was released from inhibition and exhibited an increase in responses.

Any observed increase in OTid responses quantified the amount of suppression due to Imc onto the OTid location, thereby directly estimating the strength of net “other” inhibition: % change in OTid responses = 100* (responses in Imc intact condition − responses in Imc off condition)/responses in Imc off condition. To obtain an accurate estimate of the % change, we considered the responses to stimulus S1 (in the two conditions) at all locations inside the RF as follows. We fit a straight line to the plot of OTid responses to S1 in the Imc intact vs. Imc of conditions (Fig. 3F), calculated the slope of the best-fit line, and used it to compute the average value of % change as: %change in OTid responses = 100*(slope-1). This procedure was also used to quantify “self” inhibition, “gap” inhibition and “different lobe” inhibition (below).

2. “Self” inhibition:
  To measure the strength of “self” inhibition sent from an Imc neuron to a matched location in the OTid space map, we simultaneously recorded from the Imc neuron (with a multi-barrel glass electrode filled with the kynurenic acid), and a spatially aligned “self” OTid neuron (with a tungsten electrode) as described below; we ensured that the half-max of the RF of the OTid neuron overlapped with the half-max of the Imc RF.
  We recorded a spatial tuning curve centered at the peak of the OTid (and therefore, Imc) RF. The loom speed of the stimulus used was 9.6°/s; this stimulus drives both the OTid and the Imc neuron.
  We compared the responses of the OTid neuron in the Imc-intact (baseline) and Imc inactivated condition and the difference quantified the strength of “self” inhibition (Fig. 3L, blue).
  To measure the amount of Imc inactivation, we compared the responses of the Imc neuron in the baseline and the inactivation condition as before. Kynurenic acid was able to effectively shut down Imc responses: (median strength of Imc inactivation = 92%, 95% CI of median = [86% 98%], *p* = 7.5e-9, sign test).
3. “Gap” inhibition:
  To measure the strength of “gap” inhibition sent from an Imc neuron with a multilobed RF to the locations in the OTid space map between the RF lobes (gaps), we simultaneously recorded from the Imc neuron (with a multi-barrel glass electrode filled with the kynurenic acid), and “gap” OTid neuron (with a tungsten electrode), the RF (half-max) of which was located in the gap between the half-max of the lobes of the Imc RF (Fig. 4A–C). We recorded the same set of curves as in the “other” inhibition case (see above) from the gap OTid neuron, and performed similar analyses on the data to quantify the strength of “gap” inhibition.
  As pointed out in the Neurophysiology subsection above, microiontophoresis is a focal technique for manipulating neural activity. This minimizes concerns about potentially broad spread of kynurenic acid in this experiment causing inactivation of Imc neurons encoding for the gap location as well. Notably, even if such widespread inactivation were to occur, it would not affect our interpretations for the following reason. Consider that an Imc neuron that encodes for the gap location (at which stimulus S1 is presented; Fig. 4C) is also inactivated by kynurenic acid iontophoresis to the portion of Imc encoding for stimulus S2. As demonstrated in Fig. 3, this Imc neuron does not send any inhibition to the OTid neuron encoding for S1 (i.e., to a “self” site in OTid; Fig. 3G–L). In other words, the donut-like pattern of inhibition established in Fig. 3 ensures that the strength of OTid inhibition measured by applying kynurenic acid to the portion of Imc encoding for S2, is unaffected by whether or not Imc neuron(s) encoding for S1 are also inactivated.
4. “Different lobe” inhibition:

To measure the strength of inhibition sent “from” locations within one lobe of an Imc neuron with a multilobed RF to the locations in the OTid space map within other lobes of the Imc RF (“different lobe” inhibition), we simultaneously recorded from a multilobe Imc neuron (with a multi-barrel glass electrode filled with the kynurenic acid), and an OTid neuron (with a tungsten electrode), the RF (half-max) of which overlapped with one of the lobes of the Imc RF (Fig. 4G–I). Then we measured the same set of curves as in the “other” case while ensuring one additional detail. We recorded the TC curve (curve a) with stimulus (S1) centered at the OTid RF peak (and therefore within one of the lobes of the Imc RF as well). For the TCC (curve b), the second stimulus S2 was centered at the peak of a different lobe of the Imc neuron’s multilobe RF. Thus, both stimuli excited the Imc neuron (during curve b) as they lay within its RF, but only S1 excited the OTid neuron and S2 served as a (distant) competitor from the its perspective.

An OTid neuron in this configuration was defined as a “different lobe” OTid neuron; the half max of its RF overlapped with the half max of one of the lobes of the Imc RF, but not of other lobes. (Note that by definition, every “different lobe” OTid neuron is also a potentially “self” OTid neuron, and admits to the use of the appropriate stimulus protocol to measure “self” inhibition. However, the opposite is not true, since “self” OTid neurons can be identified for Imc neurons with single lobed RFs as well, but “different lobe” OTid neurons are only defined for multilobe Imc neurons).

We applied similar analyses as in the “other” case to the data from “different lobe” OTid neurons and quantified the strength of “different lobe” inhibition.

II. Paired OTid and Ipc data collection (Figs. 5 and S4)

To test if the donut-like inhibitory motif is required for (robustness-to-noise and) categorization, we made paired recordings at spatially aligned Ipc and OTid neurons. We used a strength morphing stimulus protocol described in previously published work^8^. Briefly, we presented one stimulus (S1) inside the RF of the OTid neuron (and also Ipc neuron because their RFs overlap). Simultaneously, we presented a competing stimulus (S2) 30˚ away along azimuth from S1. As the strength of the S1 decreased, the strength of S2 stimulus increased (Fig. 5E).

We applied the same analyses described above for the model in Fig. 2, S1 to compute the categorization index for experimental data reported in Fig. 5.

### Data analyses and statistical tests

All analyses were carried out with custom MATLAB code. Parametric or non-parametric statistical tests were applied based on whether the distributions being compared were Gaussian or not, respectively (Lilliefors test of normality). The Holm-Bonferroni correction was used to account for multiple comparisons; the abbreviation “with HBMC correction” in the text stands for “with Holm-Bonferroni correction for multiple comparisons”. All tests were two-sided. Data shown as a ± b refer to mean ± standard deviation, unless specified otherwise. The “*” symbol indicates significance at the 0.05 level (after corrections for multiple comparisons, if applicable). Correlations were tested using Pearson’s correlation coefficient (*corr* command in MATLAB with the “Pearson” option).

### Reporting summary

Further information on research design is available in the Nature Research Reporting Summary linked to this article.

## Supplementary information

Supplementary Information

Supplementary Dataset

Reporting Summary

## Data Availability

Data supporting the findings of this study are available in the Zenodo repository^94^ 10.5281/zenodo.6253293.

## Code availability

The software code that supports the findings of this study are available in the Zenodo repository^94^ 10.5281/zenodo.6253293.
