# Supplementary material for: Donut-like organization of inhibition underlies categorical neural responses in the midbrain
Source: Nat Commun. 2022 Mar 30;13:1680. doi: 10.1038/s41467-022-29318-0 (PMC8967821; doi:10.1038/s41467-022-29318-0)
Supplement: Supplementary file 1 — Supplementary Information [file 41467_2022_29318_MOESM1_ESM.docx]

**SUPPLEMENTARY INFORMATION**

Donut-like organization of inhibition underlies categorical neural responses in the midbrain

**Nagaraj R. Mahajan^1^ and Shreesh P. Mysore^2,3^***


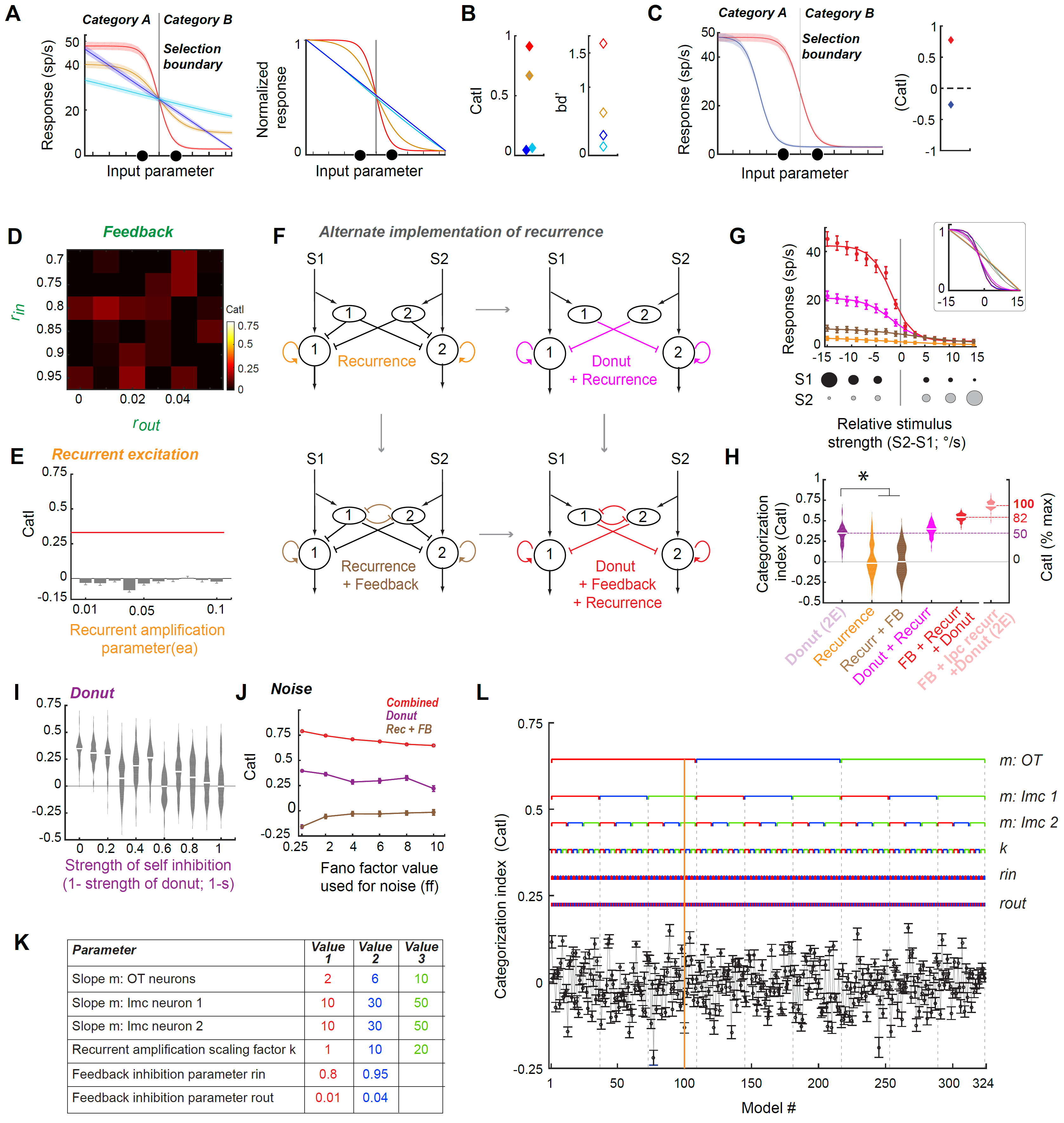


***Fig. S1. Donut-like inhibition surpasses other circuit motifs in its ability to generate categorical representations over a range of values of key model parameters and amounts of response noise (modeling).***

***(A)*** *Left: Schematic showing 4 different mathematically generated response profiles, as a function of continuously varying input; conventions as in Fig. 1A. Red and orange profiles are both categorical; orange transitions less abruptly than red, and additionally, orange is a scaled down version of red. Blue and cyan profiles are both linear (equally non-categorical); cyan is a scaled down version of blue. Right: Response profiles in left panel, normalized between 0 and 1; only means are shown. Red profile is clearly seen to transition more abruptly than orange, blue and cyan profiles are clearly seen to transition equally gradually (in a linear manner).* ***(B)*** *Left (filled symbols): Categorization index (CatI) for response profiles in A (Methods). It is sensitive to the abruptness of the transition in response profiles – it is greater for red than orange, and they are both greater than blue. Notably, CatI is insensitive to scaling – it is nearly equal for blue and cyan. Right: By contrast, discriminability computed across the selection boundary, bd’, is confounded by scaling – it is greater for blue than cyan (and the difference between its values for red and orange is greater than for CatI, due to the scaling factor between red and orange profiles in A, left). It is therefore not a reliable metric of categorization.* ***(C)*** *Left: Schematic comparing two mathematically generated sigmoidal response profiles as a function of a continuously varying input; conventions as in Fig 1A. Red – response profile in which the response transition from high to low levels occurs at the selection (or category) boundary, over a small range of input values. Dark blue – a similarly abrupt response profile in which the response transition occurs far from the selection boundary. Gray vertical line: ideal selection (or category) boundary. Translucent band: variability in responses; fano factor of 6 used to generate these responses (Methods). Right: Categorization index (CatI) characterizes strength of categorization of response profiles in left panel. CatI value is positive and high for response profiles (red) that exhibit an abrupt transition across the ideal boundary. However, it is negative for response profiles (blue) that exhibit a transition far from the boundary (even when the transition is abrupt), because the within category discriminability (WCD) is higher than the between category discriminability (BCD)).* ***(D)*** *Effect of varying strength of feedback inhibition in model in Fig. 2A middle-column top, on CatI. r_in_: input divisive factor; r_out_: output divisive factor (Methods). Range of variation based on previously published work^44^. Maximum CatI = 0.157 (lower than from the donut-like motif, CatI = 0.331).* ***(E)*** *Effect of varying strength of recurrent amplification in model in Fig. 2A, left column-bottom on CatI. Red line: value from circuit with donut-like motif only. CatI does not change systematically (corr=* *-0.14; p =0.54; two-sided Student's t test), indicating that varying the strength of amplification does not affect the CatI estimate for this motif in Fig. 2D.* *mean ± s.e.m; n=50 model neurons.* ***(F-H)*** *Computational models with alternate implementation of Ipc-recurrent amplification, and termed ‘recurrent amplification’ (similar to that employed in the modeling literature). Here, unlike Ipc-recurrent amplification in Fig. 2A, recurrent amplification within each channel is not under the control of powerful competitive inhibition. Nonetheless, just like in Fig. 2D, recurrent amplification by itself, or in conjunction with feedback, is ineffective at producing categorization (i.e., CatI values are low). (F) Model circuits with recurrence alone (top-left), recurrence and donut (top-right), recurrence and feedback (bottom-left), and all three motifs (bottom-right). (G) Plots of the response profiles to strength-morphing protocol (and normalized response profiles; inset), obtained from the four model circuits in F. (H) Plots of CatI of these response profiles. All conventions as in Fig. 2ACD; center lines in the violin plots indicate median values.* ***(I)*** *Effect of varying strength of ‘self’- inhibition in model in Fig. 2A, left column-top on CatI. Corr = -0.81, p = 3e-3 (two-sided Student's t test). Maximal effect on CatI is when ‘self’-inhibition=0, i.e., when the circuit has donut-like inhibition; n=50 model neurons; center lines in the violin plots indicate median values.* ***(J)*** *Comparison (across three key models) of CatI from simulated response profiles as a function of fano-factor. Models are: circuit with donut-like inhibitory motif only (Fig. 2A, middle column-second from top), circuit with Ipc-recurrent amplification and feedback inhibition together (Fig. 2A, middle column-second from bottom), and circuit with all three motifs combined (Fig. 2A, right column-bottom).* ***(K, L)*** *Exploration of the efficacy of models without the donut-like motif (but with feedback inhibition and recurrent amplification) in achieving categorical responses, when values of various key model parameters are varied. The model architecture used in these simulations is the same as that in F, bottom-left****.*** *(K)* *Table showing parameters that are varied and their values; color codes signify different values of each parameter. A total of 324 combinations of the parameter values are explored. (L) CatI of response profiles obtained from each of these 324 circuit models; mean ± s.e.m, n=50 model neurons. Each point in the plot corresponds to a combination of parameter values. The brackets above the CatI plot indicate the color-coded value that each parameter takes for that model; the color codes are consistent with the table in (K); vertical dashed lines have been added to aid visualization. Vertical orange line highlights model #100; for this model, m (OT) = 2 (red), m (Imc1) =50 (green), m (Imc2) = 50 (green); k = 1 (red); r_in_ =0.95 (blue); r_out_ =0.04 (blue).*

***Fig. S2. Supporting experimental data for Figure 3. (A)*** *Recovery of OTid responses from kynurenic acid iontophoresis for experiments in Fig. 3. Data on left: “Other” experiments. OTid responses revealed significant change during iontophoresis (red dots; subset of the data reproduced from Fig. 3L, n = 10), but returned to pre-drug baseline (horizontal line) in recovery (black dots; p=0.32, t-test against 0). Recovery data obtained 15 min after iontophoretic eject current was switched to retain current; Methods). Data show recovery, demonstrating that the effects reported Fig. 3 are due specifically to drug iontophoresis/Imc inactivation. Data on right: “Self” experiments. OTid responses showed no significant change during iontophoresis (blue dots; reproduced from Fig. 3L, n = 12), and stayed around zero in recovery (black dots; p=0.58, two-sided t-test against 0).* ***(B)*** *“Other” experiment. Comparison of suppression provided by Imc with that due to S2 (i.e., the maximum amount of suppression experienced by the OTid neuron in this stimulus protocol). This is done by comparing (i) % suppression of OTid responses by stimulus S2 when Imc is intact, computed by comparing OTid responses to S1 alone versus the paired presentation of S1 and S2 (teal^49^), with (ii) % suppression of OTid responses to paired presentation of S1 and S2 produced by Imc inactivation, computed by comparing OTid responses in the Imc-intact to Imc-off conditions (red; data reproduced from Fig. 3L). Consistent with^32^, nearly all the suppression due to competitor S2 (teal) is supplied by Imc (red) (p= 0.22, two-sided t-test, teal vs. red, n = 19), verifying that Imc supplies powerful “other” inhibition. Box plot conventions as in Fig. 3L.* ***(C)*** *Quantifying the effectiveness of Imc inactivation by kynurenic acid iontophoresis in the “other” (red) and “self”-inhibition (blue) experiments. In both cases, the inactivation was highly effective. Red: median % change in response; median = 95%, 95% CI of median = [87%, 103%], *: p<0.05, p = 3.8 e-6, two-sided sign test, n = 19; Blue: median % change in responses median =0.92, 95% CI of median = [86%, 98%], p = 7.45e-9, two-sided sign test, n = 28. Box plot conventions as in Fig. 3L.*


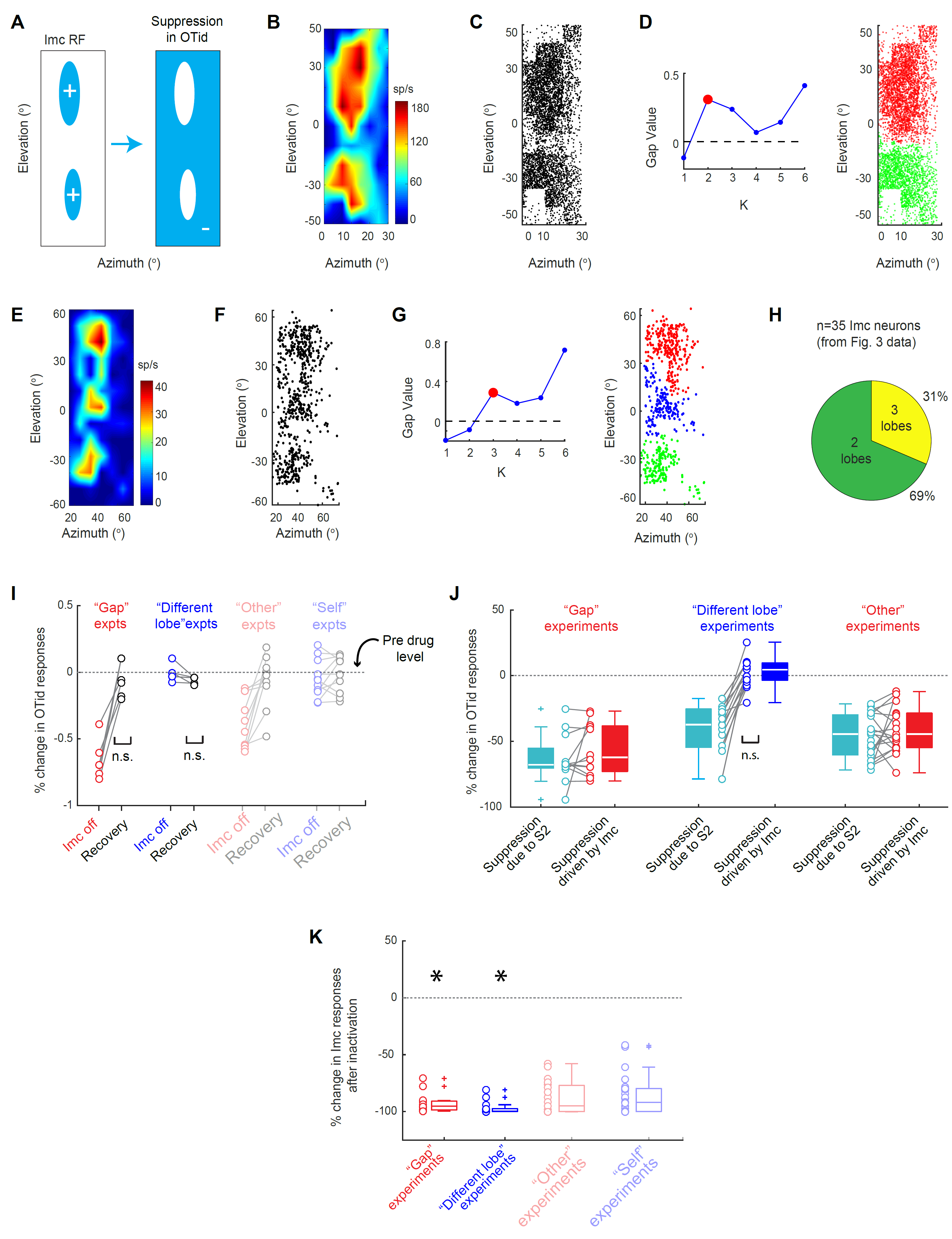


***Fig. S3. Detecting the number of Imc RF lobes, and other supporting experimental data for Figure 4.***

***(A)*** *Schematic representation of a multi-holed donut. Left: Two-lobed RF of a putative Imc neuron (left; blue ovals are the RF lobes). Right: Pattern of inhibition created by this neuron in the OTid such that it spares all the locations form which it receives input. This complementary output projection pattern results in two “holes” – areas where no inhibition arrives due to the neuron on the left.* ***(B)*** *Imc RF reproduced from Fig. 4A-left.* ***(C)*** *Firing rate map from B converted to density of points in 2-D plane following published procedures^52^.* ***(D)*** *Left: Density peaks clustering method^95^ is applied to the data in B, forcing the method to yield either 1 cluster, 2 clusters, … up to 6 clusters. Following that, the gap statistic model selection metric^96^ is applied to the clustering results to identify the optimal number of clusters in the data. The optimal number is the number for which the “gap value” exceeds 0 for the first time; here it is 2. Right: Same as B, but with the two colors indicating the two distinct lobes identified by the clustering method (+ gap statistic) as the optimal two best clusters in the RF data.* ***(E-G)*** *Same as B-D, but for Imc RF in Fig. 4G-left; determined to be three-lobed.* ***(H)*** *Pie-chart showing proportion of two-lobed vs. three-lobed RFs across all the multi-lobed Imc neurons recorded in these experiments (Fig. 4).* ***(I)*** *Recovery of OTid responses from kynurenic acid iontophoresis for experiments in Fig. 4. Conventions as in Fig. S2A; data from Fig. S2A reproduced here for comparison. Data show recovery (“Gap” experiment: p =0.39, n = 6, two-sided t-test of black data against 0; “Other” lobe experiment: p=0.29, n = 6, two-sided ranksum test of black data against 0), demonstrating that any effects reported Fig. 4 are due specifically to drug iontophoresis/Imc inactivation.* ***(J)*** *Comparison of suppression provided by Imc with that due to S2 (i.e., the maximum amount of suppression experienced by the OTid neuron in this stimulus protocol) in the “gap” (red data) and “different lobe” (blue data) experiments. Conventions as in Fig. S2B; data from Fig. S2B reproduced here for comparison. “Gap” experiment: Consistent with^32^, nearly all the suppression due to competitor S2 (teal) is supplied by Imc (red) (p= 0.47, n = 12, teal vs. red, two-sided ranksum test). “Different lobe” experiment: The Imc neuron that is being inactivated does not provide any of the inhibition to the OTid neuron due to S2 (p = 0.35, n = 17, blue dots against 0, two-sided t-test). This clearly demonstrates, consistent with the predictions of ^52^, that a different Imc neuron exists, which has one RF lobe encoding S2’s location, but other RF lobes (if there are others for that neuron) not encoding S1’s location, thereby delivering inhibition to that location. This is one of the ‘signature’ properties for the combinatorial encoding of space described in that study. Box plot conventions as in Fig. 3L.* ***(K)*** *Quantifying the effectiveness of Imc inactivation by kynurenic acid iontophoresis in the “gap” (red) and “different lobe” (blue) experiments. “Gap” experiment: ‘*’: p < 0.05, p =1.13 e-12 two-sided t-test against 0, n = 12; “Other” lobe experiment: p =1.53e-5, two-sided signtest, n = 17. Conventions as in Fig. S2C; data from Fig. S2C reproduced here for comparison. Box plot conventions as in Fig. 3L.*

******

***Fig. S4. Supporting modeling and experimental results for Figure 5.***

***(A-C) Modeling: Effect of silencing recurrent amplification.****(A) Computational model of modified version of the midbrain selection network; reproduced from Fig. S1F, bottom-right. Recall that this network has donut-like inhibition, feedback inhibition, and recurrent amplification (just like the midbrain selection network), but with the one modification that the recurrent amplification (black curved arrow) is not under the control of competitive inhibition. The strength of curved black arrow is not affected by inhibition from oval neurons, in contrast to ‘Ipc-recurrent’ amplification in the midbrain selection network, which is (Fig. 5A). Incidentally, this form of recurrence, termed simply, ‘recurrent’ amplification, represents the typical implementation in published models of selection. (B) Model in A, but with recurrent amplification silenced. (C) Plots of CatI of the response profiles obtained from model circuits in A (red-gray) and B (blue) presented with the strength-morphing protocol (n=50 model neurons; center lines in violin plots indicate median values). These results show that silencing just recurrent amplification (when it is not under the control of competitive inhibition, B), causes no discernible impact on categorization (C, blue vs. red-gray). (This is in contrast to inactivation of Ipc in the barn owl midbrain network, which causes abolishment of categorization, just as introducing self-inhibition or ‘filling-in’ the donut-hole does; Fig. 5D, pink vs. gold. Thus, the proposed focal Ipc inactivation in the barn owl midbrain network (Fig. 5C) is akin to introducing self-inhibition onto Ipc (Fig. 5B) rather than to simply silencing recurrent amplification.) All conventions as in Fig. 5.* ***(D-E) Modeling: Validity of inactivating Ipc (“filling-in the donut-hole”) only in channel #1 in Figure 5C.****(D) Left: Schematic of OT-Imc-Ipc circuit in barn owl midbrain; reproduced from Figure 5A. In this circuit, feedback connections between the Ipc and OT are focal and spatially-specific: there are no connections between the Ipc neuron encoding one stimulus and the OTid neuron encoding the other. Right: Inactivation of Ipc neuron encoding stimulus S2. We hypothesized that because of the specialized connectivity in this midbrain circuit, inactivation of Ipc neuron encoding stimulus S2 should not impact the responses of the OTid neuron encoding stimulus S1. (E) Simulated responses of OTid neuron encoding stimulus S1 to the strength-morphing protocol, without (pink; D-left) and with (green; D-right) inactivation of Ipc neuron encoding S2; responses are identical in both conditions. In other words, not inactivating Ipc neuron encoding S2 has no impact on the competitive responses measured at OTid neuron encoding S1. Thus, our experimental approach of “closing the hole” for only the channel that encodes for S1 presents no confounds to interpretation.****(F) Experiments: Ipc is effectively inactivated in experiments in Fig. 5.****Mean suppression of Ipc responses upon iontophoresis of kynurenic acid was 77.42% with a standard deviation of 22.82%; p-value = 5.3e-7; n = 11; two-sided t-test against 0. Box plot conventions as in Fig. 3L*
